# Rad regulation of Ca_V_1.2 channels controls cardiac fight-or-flight response

**DOI:** 10.1038/s44161-022-00157-y

**Published:** 2022-11-14

**Authors:** Arianne Papa, Sergey I. Zakharov, Alexander N. Katchman, Jared S. Kushner, Bi-xing Chen, Lin Yang, Guoxia Liu, Alejandro Sanchez Jimenez, Robyn J. Eisert, Gary A. Bradshaw, Wen Dun, Shah R. Ali, Aaron Rodriques, Karen Zhou, Veli Topkara, Mu Yang, John P. Morrow, Emily J. Tsai, Arthur Karlin, Elaine Wan, Marian Kalocsay, Geoffrey S. Pitt, Henry M. Colecraft, Manu Ben-Johny, Steven O. Marx

**Affiliations:** 1 Division of Cardiology, Department of Medicine, Columbia University, Vagelos College of Physicians and Surgeons, New York, NY, USA.; 2 Department of Physiology and Cellular Biophysics, Columbia University, Vagelos College of Physicians and Surgeons, New York, NY, USA.; 3 Department of Systems Biology, Laboratory of Systems Pharmacology, Harvard Medical School, Boston, MA, USA.; 4 Institute for Genomic Medicine, Columbia University, Vagelos College of Physicians and Surgeons, New York, NY, USA.; 5 Department of Biochemistry and Molecular Biophysics, Vagelos College of Physicians and Surgeons, New York, NY, USA.; 6 Cardiovascular Research Institute and Department of Medicine, Weill Cornell Medical College, New York, NY, USA.; 7 Department of Pharmacology and Molecular Signaling, Columbia University, Vagelos College of Physicians and Surgeons, New York, NY, USA.; 8 Present address: Department of Experimental Radiation Oncology, University of Texas MD Anderson Cancer Center, Houston, TX, USA.; 9 These authors contributed equally: Arianne Papa, Sergey I. Zakharov, Alexander N. Katchman, Jared S. Kushner.

## Abstract

Fight-or-flight responses involve β-adrenergic-induced increases in heart rate and contractile force. In the present study, we uncover the primary mechanism underlying the heart’s innate contractile reserve. We show that four protein kinase A (PKA)-phosphorylated residues in Rad, a calcium channel inhibitor, are crucial for controlling basal calcium current and essential for β-adrenergic augmentation of calcium influx in cardiomyocytes. Even with intact PKA signaling to other proteins modulating calcium handling, preventing adrenergic activation of calcium channels in Rad-phosphosite-mutant mice (4SA-Rad) has profound physiological effects: reduced heart rate with increased pauses, reduced basal contractility, near-complete attenuation of β-adrenergic contractile response and diminished exercise capacity. Conversely, expression of mutant calcium-channel β-subunits that cannot bind 4SA-Rad is sufficient to enhance basal calcium influx and contractility to adrenergically augmented levels of wild-type mice, rescuing the failing heart phenotype of 4SA-Rad mice. Hence, disruption of interactions between Rad and calcium channels constitutes the foundation toward next-generation therapeutics specifically enhancing cardiac contractility.

All vertebrates exhibit the ‘fight-or-flight’ response to immediate stress. The ability of the heart to beat faster and more powerfully is central to this evolutionarily conserved survival instinct. Under resting conditions, only a fraction of heart pumping power is utilized, but, during stress or exercise, the heart rate and force of contraction increase. Norepinephrine released from sympathetic fibers innervating the heart and epinephrine secreted by adrenal chromaffin cells bind to cardiac β-adrenergic receptors, initiating an intracellular signaling cascade that generates cyclic (c)AMP and activates protein kinase A (PKA), the targets of which control intracellular calcium concentration in cardiomyocytes^1^.

The critical PKA targets for augmenting intracellular Ca^2+^ concentration and cardiac contractility have remained elusive. PKA phosphorylation of phospholamban (PLN) and subsequent release of PLN-induced inhibition of the sarcoplasmic reticulum (SR) Ca^2+^ pump (SERCA) increases the rate of relaxation^2^ and modestly contributes to increasing the Ca^2+^ transient amplitude by ~30%^3–5^. PKA phosphorylation of SR ryanodine receptor (RyR2) channels increases the RyR2 open probability^6,7^, although the role of augmented SR Ca^2+^ release^8^ in sustaining increased cardiac contractility is controversial^9^.

Voltage-gated Ca^2+^ channels (Ca_V_) are also activated by PKA^10–12^. It is now clear that the long-held assumption of β-adrenergic enhancement of Ca_V_1.2 involving direct phosphorylation of the Ca_V_1.2 pore-forming α_1_- and/or auxiliary β-subunits is incorrect: β-adrenergic agonists still upregulate current through Ca_V_1.2 in which all potential phosphorylation sites on the α- and β-subunits have been removed^13–15^. We recently identified the small RGK G-protein Rad, an inhibitor of voltage-activated Ca^2+^ channels^16,17^, as an alternative target^15^. Using an ascorbate peroxidase (APEX2)-catalyzed proximity labeling in transgenic mouse hearts, combined with quantitative proteomics, we observed that, under basal conditions, Rad was enriched in the neighborhood of Ca_V_1.2; on exposure to a β-adrenergic agonist, however, Rad was depleted from around Ca_V_1.2 (ref.^15^). With the identification that PKA phosphorylation of the small RGK G-protein Rad relieves its inhibition of heterologously expressed, voltage-gated Ca^2+^ channels^17^ and the development of mice expressing mutant Rad and Ca_V_1.2 channels, the mechanisms underlying the primordial fight-or-flight responses that boost heart rate and contractility can now be ascertained.

## Results

### Adrenergic regulation of calcium influx

**Extended Data Fig. 1 |:**
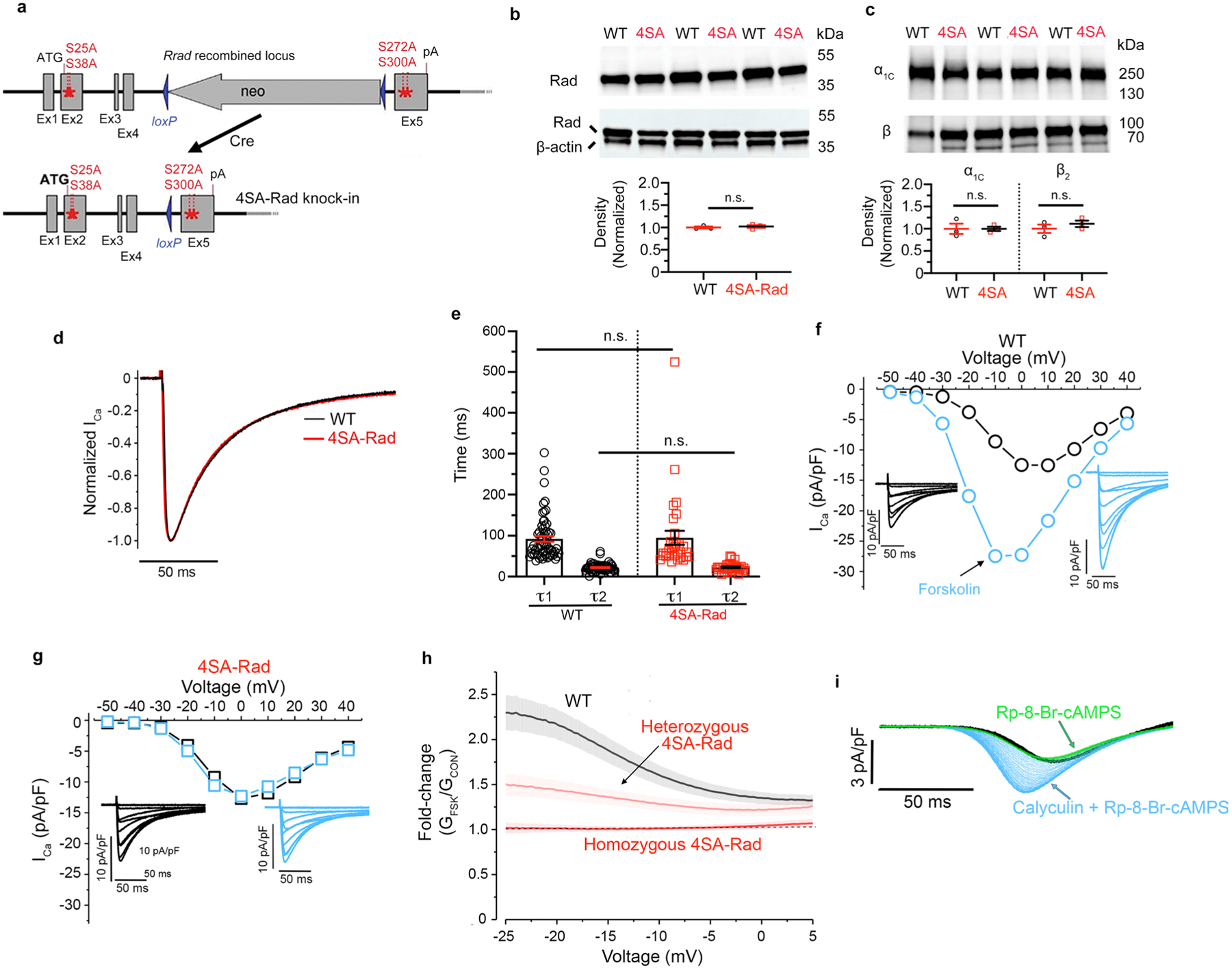
Rad phosphorylation is required for augmentation of Ca^2+^ channel current. **a**, Schematic depicting creation of 4SA-Rad mice by homologous recombination. Alanine-substitutions were introduced for four serine (Ser) residues, Ser^25^ and Ser^38^ in exon2 (Ex2), and Ser^272^ and Ser^300^ in exon5 (Ex5). The neomycin cassette was removed by Cre recombinase. **b**, Anti-Rad and anti-β-actin antibody immunoblots. Lower: Graph of quantification of intensity of Rad band normalized to β-actin loading control. Data are mean ± s.e.m.; *P* = 0.61 by unpaired two-tailed *t*-test. N = 3 mice in each group. **c**, Anti-α_1C_ and anti-β subunit antibody immunoblots of WT and 4SA-Rad ventricular cardiomyocyte lysates. Lower: Graph of quantification of intensity of α_1C_ and β subunit bands normalized to β-actin. Data are mean ± s.e.m.; *P* = 0.99 and *P* = 0.41, from left to right, by unpaired two-tailed *t*-test. N = 3 mice in each group. **d**, Exemplar traces of normalized Ca^2+^ current in response to a step depolarization to +10 mV. **e**, Graph of τ_1_ and τ_2_ inactivation. Fits were obtained using two exponentials by Clampfit. Data are mean ± s.e.m.; *P* = 0.86 for τ_1_ and *P* = 0.77 for τ_2_ by unpaired two-tailed *t*-test. n = 61 WT cells and 31 4SA-Rad cells from 22 and 8 mice respectively. **f-g**, Exemplar current-voltage relationships of Ca^2+^ channels from WT (**f**) and 4SA-Rad cardiomyocytes (**g**) acquired in the absence (black trace) and presence of 10 μM forskolin (blue trace). Insets: Exemplar whole-cell Ca_V_1.2 currents. Pulses from −60 mV to +50 mV before (black traces) and 3 minutes after (blue traces) forskolin. **h**, Ratio of Ba^2+^ current after forskolin to Ba^2+^ current before forskolin for WT, heterozygous 4SA-Rad, and homozygous 4SA-Rad cardiomyocytes. Data are mean ± s.e.m.; n = 25 WT cells from 6 mice; 26 heterozygous 4SA-Rad cells from 3 mice; 28 homozygous 4SA-Rad cells from 5 mice. **i**, Ba^2+^ current elicited by voltage ramp every 6 seconds, with black traces (control), green traces after PKA inhibitor Rp-8Br-cAMPS, and blue traces after calyculin A in presence of Rp-8Br-cAMPS in WT ventricular myocytes. Representative of 4 similar experiments.

**Fig. 1 |:**
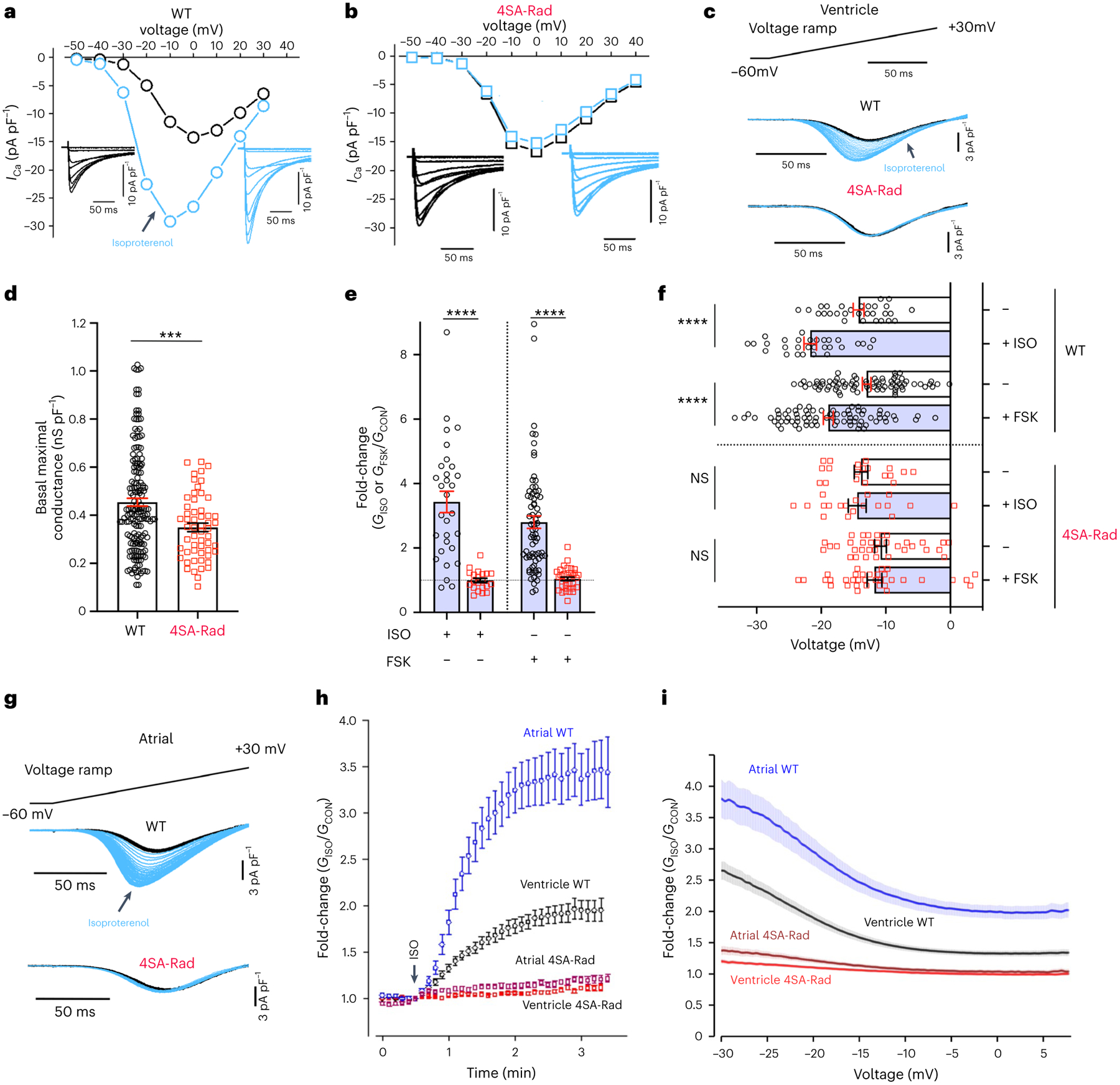
Adrenergic agonist-induced stimulation of Ca^2+^ current requires phosphorylation of Rad in ventricular and atrial cardiomyocytes. **a**,**b**, Exemplar current–voltage relationships of Ca^2+^ channels from WT and 4SA-Rad cardiomyocytes in the absence (black trace) and presence (blue trace) of isoproterenol (ISO). Inset: exemplar whole-cell Ca_V_1.2 currents. Pulses from −60 mV to +50 mV before (black traces) and 3 min after (blue traces) 200 nM isoproterenol. **c**, Ba^2+^ current elicited by voltage ramp from −60 mV to +30 mV, with black traces obtained before and blue traces after isoproterenol in WT and 4SA-Rad ventricular myocytes. **d**, Graph of maximal conductance density (at +10 mV) for WT (172 cells, 22 mice) and 4SA-Rad cardiomyocytes (56 cells, 11 mice), acquired via the step protocol. Data are the mean ± s.e.m. ***P < 0.001 by unpaired, two-tailed Student’s *t*-test. Specific *P* values can be found in the associated Source data (see Supplementary Information). **e**, Fold-change in peak current at −20 mV caused by isoproterenol or forskolin (FSK). Data are mean ± s.e.m. ****P < 0.0001 by unpaired, two-tailed Student’s *t*-test. WT: isoproterenol, 30 cells, 8 mice and forskolin, 71 cells, 15 mice; 4SA-Rad: isoproterenol, 21 cells, 4 mice and forskolin, 35 cells, 7 mice. **f**, *V*_50_ before and after isoproterenol or forskolin. Data are the mean ± s.e.m. ****P < 0.0001 for WT, *P* = 0.67 (isoproterenol), *P* = 0.41 (forskolin) for 4SA-Rad by unpaired, two-tailed Student’s *t*-test. NS, not significant. WT: isoproterenol, 30 cells, 8 mice and forskolin, 76 cells, 15 mice; 4SA-Rad: isoproterenol, 20 cells, 4 mice and forskolin, 36 cells, 7 mice. **g**, Same as **c** except with atrial cardiomyocytes. **h**, Diary plot of normalized fold-change of conductance at −20 mV for WT and 4SA-Rad atrial and ventricular myocytes. Data are mean ± s.e.m. Ventricle WT: 22 cells, 3 mice; 4SA-Rad: 31 cells, 3 mice; atrial WT: 29 cells, 3 mice; 4SA-Rad: 20 cells, 3 mice. **i**, Graph of ratio of Ba^2+^ conductance after to Ba^2+^ conductance before isoproterenol treatment versus voltage. Data are mean ± s.e.m. Ventricle WT: 29 cells, 3 mice; 4SA-Rad: 35 cells, 3 mice; atrial WT: 34 cells, 3 mice; 4SA-Rad: 29 cells, 3 mice.

We developed knock-in mice (Extended Data Fig. 1a) in which the four evolutionarily conserved PKA-phosphorylated serine residues of the endogenous murine *Rrad* locus (Ser25, Ser38, Ser272 and Ser300) were replaced by alanine residues (4SA-Rad). The protein expression in cardiomyocytes of Rad (Extended Data Fig. 1b) or the principal Ca_V_1.2 subunits, α_1C_ and β_2B_, were not affected by introducing these mutations in Rad (Extended Data Fig. 1c). We interrogated the electrophysiological properties of Ca^2+^ channels in ventricular cardiomyocytes isolated from WT and homozygous 4SA-Rad knock-in mice using either traditional voltage-steps with Ca^2+^ as the charge carrier (Fig. 1a,b) or a voltage ramp protocol applied every 6 s with Ba^2+^ as the charge carrier (Fig. 1c). The ramp protocol enables the monitoring of agonist effects over time. The basal electrophysiological properties of Ca^2+^ channels in the 4SA-Rad cardiomyocytes did not differ (Extended Data Fig. 1d,e), but for the amplitude of peak current that was reduced compared with wild-type (WT) cardiomyocytes (Fig. 1d), despite unchanged protein expression of Ca_V_1.2 subunits. In sharp contrast to the Ca^2+^ current of WT ventricular cardiomyocytes, neither was the Ca^2+^ current of 4SA-Rad ventricular cardiomyocytes augmented nor was the membrane potential for half-maximum activation, *V*_50_, shifted by either the nonselective β-adrenoreceptor agonist isoproterenol or the adenylyl cyclase activator forskolin (Fig. 1a–c,e,f and Extended Data Fig. 1f–h). Isoproterenol induced a rapid increase in current across all test potentials in WT but not 4SA-Rad ventricular cardiomyocytes (Fig. 1h,i). Cardiomyocytes isolated from heterozygous 4SA-Rad knock-in mice, which bear one WT allele and one 4SA-Rad allele, demonstrated an intermediate response to forskolin (Extended Data Fig. 1h).

Cardiac atrial contraction normally accounts for ~10% of left ventricular filling at rest and up to ~40% of ventricular filling at high heart rates, such as in fight-or-flight responses. The atria in the heart respond to isoproterenol with much larger increases in developed tension, contractility and relaxation rates than ventricular papillary muscles^18^. Consistent with these findings, we observed substantially greater isoproterenol-induced augmentation of the Ca^2+^ current in atrial myocytes than in ventricular myocytes (Fig. 1g–i). As in ventricular myocytes, isoproterenol failed to increase the current in 4SA-Rad atrial myocytes (Fig. 1g–i). Thus, adrenergic augmentation of cardiac Ca^2+^ currents in both atrial and ventricular myocytes is fully dependent on phosphorylation of Rad and not on phosphorylation of the principal channel α_1C_- and β_2_-subunits^13–15^.

### Calyculin A-induced changes in Ca_V_1.2 neighbors

As the balance between kinase and phosphatase activity contributes to setting the basal Ca^2+^ current in cardiomyocytes, which was reduced in 4SA-Rad cardiomyocytes (Fig. 1d), we set out to identify key components underlying basal Ca_V_1.2 regulation. Application of various protein phosphatase inhibitors, such as okadaic acid, microcystin or calyculin A, to cardiomyocytes results in large increases in Ca^2+^ current amplitude^19–23^ via an unknown mechanism. In mice, the calyculin A-induced increase in current in WT cardiomyocytes is insensitive to the PKA inhibitor Rp-8-Br-cAMPS (Extended Data Fig. 1i)^20,23^.

**Fig. 2 |:**
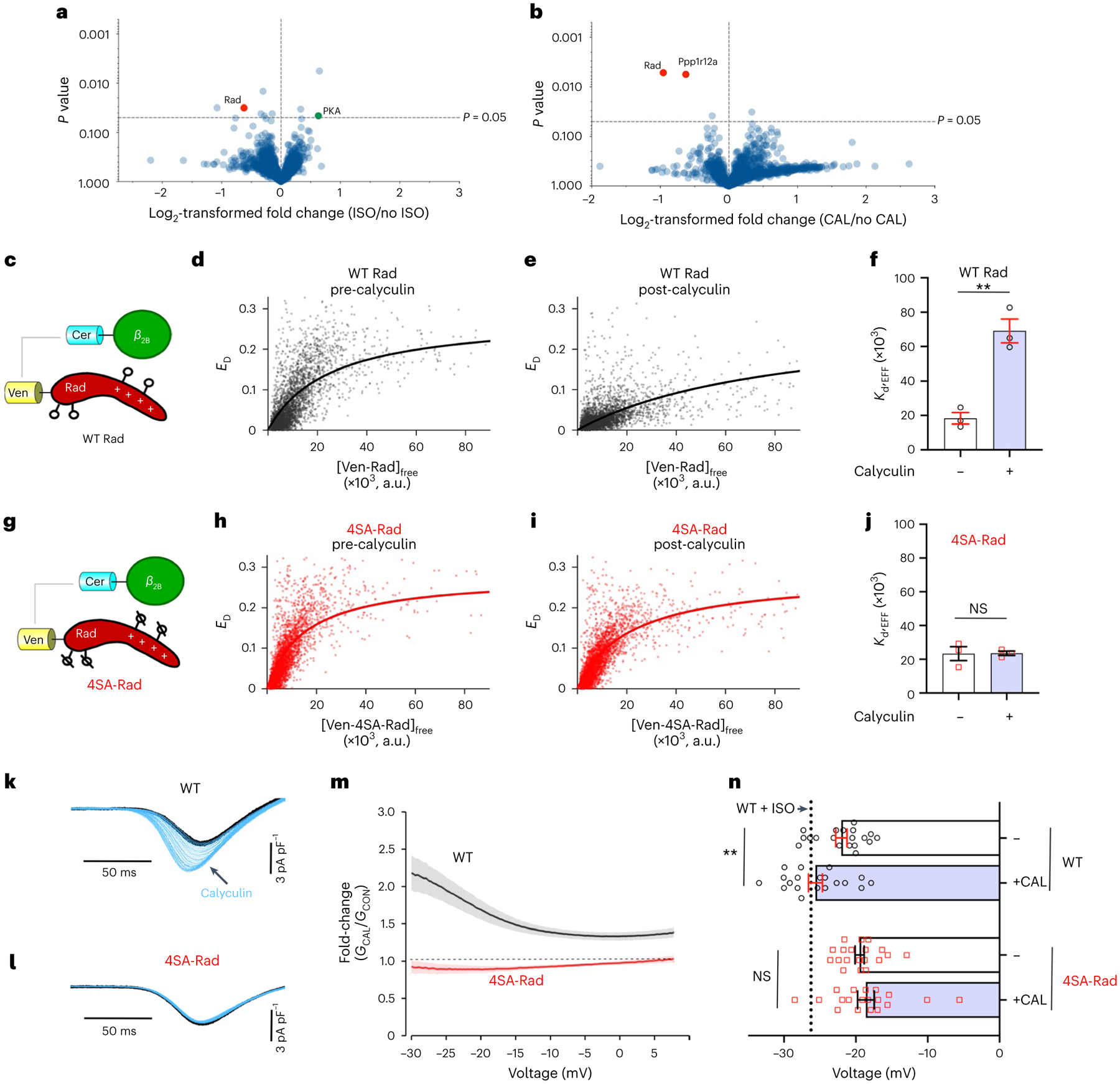
Augmentation of Ca^2+^ influx by phosphatase inhibitors is Rad dependent. **a**,**b**, Volcano plots of fold-change for relative protein quantification by TMT MS of α_1C_-APEX2 cardiomyocytes treated with vehicle, isoproterenol or calyculin A (CAL). The data shown are means for five pairs of biologically independent samples. **a**, Comparison between isoproterenol-treated and vehicle. **b**, Comparison between calyculin A and vehicle. A nonadjusted, unpaired, two-tailed Student’s *t*-test was used. PPP1R12A, protein phosphatase 1 regulatory subunit 12A. See Supplementary Table 1 for complete dataset. **c**,**g**, Schematics of the FRET pairs, Cer–β_2B_ with Ven–WT-Rad (**c**) or Ven–4SA-mutant Rad (**g**) (where Ven is the Venus tag and Cer is the Cerulean tag). **d**,**e**, FRET efficiency (*E*_D_) is plotted against the free concentration Ven–WT Rad in the absence of (**d**) and 10 min after (**e**) exposure to calyculin A. a.u., arbitrary units. Solid line fits a 1:1 binding isotherm. **f**, Graph summarizing *K*_d,EFF_ for the binding of WT Rad and the β_2B_-subunit. Data are the mean ± s.e.m. (*n* = 3 for pre- and post-calyculin). **P < 0.01 by unpaired, two-tailed Student’s *t*-test. **h**,**i**, Same as **d** and **e** except that Ven–4SA-Rad was used. **j**, Graph summarizing *K*_d,EFF_ for the binding of 4SA-Rad and the β_2B_-subunit. Data are the mean ± s.e.m. (*n* = 3 for pre- and post-calyculin; *P* = 0.96 (NS) by unpaired, two-tailed Student’s *t*-test). **k**,**l**, Ba^2+^ current elicited by voltage ramp every 6 s, with black traces obtained before and blue traces obtained after calyculin A in WT (**k**) and 4SA-Rad (**l**) ventricular myocytes. **m**, Graph of ratio of Ba^2+^ conductance after calyculin A treatment to Ba^2+^ conductance before treatment versus voltage of ventricular cardiomyocytes isolated from WT and 4SA-Rad mice. Data are mean ± s.e.m. (WT: 21 cells, 3 mice; 4SA-Rad: 19 cells, 3 mice). **n**, *V*_50_ before and after calyculin A. Black dashed line in *V*_50_ for WT + isoproterenol. Data are the mean ± s.e.m. **P < 0.01 for WT, *P* = 0.52 for 4SA-Rad by unpaired, two-tailed Student’s *t*-test. WT: 18 cells, 3 mice; 4SA-Rad: 18 cells, 3 mice.

Previously, we applied enzyme-catalyzed proximity labeling and multiplexed mass spectrometry (MS) in cardiomyocytes, which quantified isoproterenol-induced changes in the molecular environment of Ca_V_1.2 channels of cardiomyocytes^15^. We now leverage this approach to identify the mechanism by which Ca^2+^ influx can be modulated in cardiomyocytes at basal conditions. Using transgenic mice expressing Ca_V_1.2 α_1C_-APEX2 (ref.^15^) and multiplexed tandem mass tag (TMT) MS, we assessed changes in the Ca_V_1.2 neighborhood after adding calyculin A or isoproterenol. The remarkable 50% decrease in Rad level in proximity to Ca_V_1.2 caused by calyculin A exposure is comparable to the isoproterenol-induced depletion of Rad (Fig. 2a,b and Supplementary Table 1), suggesting that Rad is also involved in nonadrenergic regulation of Ca_V_1.2. In contrast to addition of isoproterenol (Fig. 2a), we did not observe recruitment of the PKA catalytic subunit to the channel neighborhood by calyculin A (Fig. 2b) despite its quantification by MS (Supplementary Table 1).

We hypothesized that the calyculin A-induced change in Rad localization may depend on Rad phosphorylation, probably on at least some of the four phosphorylated serine residues in Rad. We utilized flow cytometry Förster resonance energy transfer (FRET) two-hybrid assay^24^ to probe potential calyculin-mediated changes in the macromolecular complex of Ca_V_1.2. At baseline, robust binding is detected between Cerulean-tagged β_2B_-subunit and Venus-tagged WT Rad expressed in human embryonic kidney (HEK) cells (Fig. 2c,d,f). Incubation of HEK cells with calyculin A markedly weakened this interaction (Fig. 2e,f). Calyculin A had no effect, in contrast, on the interaction between the β_2B_-subunit and 4SA-Rad (Fig. 2g–j). As definitive proof of the mechanism by which phosphatase inhibition increases ionic currents through Ca_V_1.2 channels, we compared the effects of calyculin A in WT and 4SA-Rad ventricular myocytes. Compared with WT cardiomyocytes (Fig. 2k), calyculin A neither increased the Ca_V_1.2 current amplitude (Fig. 2l,m) nor shifted the current–voltage relationship in a hyperpolarizing direction (Fig. 2n) in the 4SA-Rad ventricular cardiomyocytes. Thus, signaling pathways other than the β-adrenergic–PKA system are integrated by Rad phosphorylation and contribute to the setting of the basal Ca^2+^ current in cardiomyocytes.

### Adrenergic augmentation of Ca^2^ transient

**Fig. 3 |:**
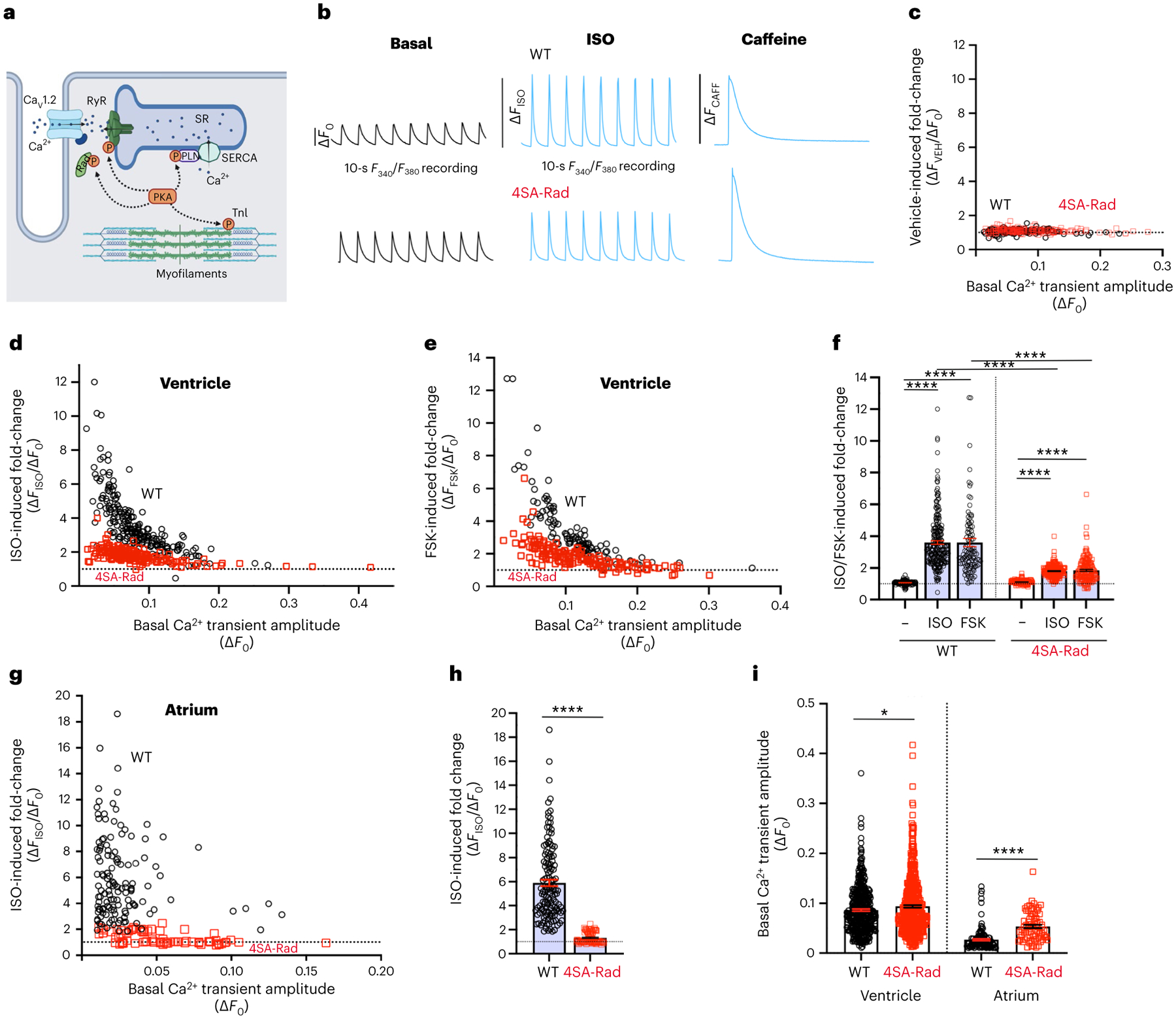
Rad phosphorylation is required for adrenergic agonist-induced augmentation of Ca^2+^ transient. **a**, Model depicting the targets of β-adrenergic agonist stimulation in cardiomyocytes. TnI: troponin I, P: phosphorylated protein. Schematic created using BioRender.com. **b**, Schematic of experimental protocol. Cardiomyocytes from WT and 4SA-Rad cardiomyocytes were loaded with Fura2-AM and subsequently field stimulated at 1 Hz. Fluorescence was acquired for 10 s before and after superfusion with vehicle, 100 nM isoproterenol (ISO) or 5 μM forskolin (FSK). SR Ca^2+^ load was determined after infusion of caffeine. **c**, Graph of fold-change after vehicle infusion of Ca^2+^ transient amplitude versus basal amplitude in WT (black) and 4SA-Rad (red) ventricular myocytes. WT: 128 cells, 3 mice; 4SA-Rad: 175 cells, 3 mice. **d**, Graph of isoproterenol-induced fold-change of Ca^2+^ transient amplitude versus basal amplitude in WT (black) and 4SA-Rad (red) ventricular myocytes. WT: 225 cells, 4 mice; 4SA-Rad: 183 cells, 5 mice. **e**, Graph of forskolin-induced fold-change of Ca^2+^ transient amplitude versus basal amplitude in WT (black) and 4SA-Rad (red) ventricular myocytes. WT: 109 cells, 3 mice; 4SA-Rad: 149 cells, 5 mice. **f**, Graph of isoproterenol- and forskolin-induced fold-change in amplitude of Ca^2+^ transient. Data are mean ± s.e.m. *P* < 0.0001 by one-way ANOVA, ****P < 0.0001 by Sidak’s multiple-comparison test (*n* = 128, 225, 109, 175, 183 and 149 cardiomyocytes from 3, 4, 3, 3, 5, 5 mice, respectively, from left to right). **g**, Same as **d** except with atrial cardiomyocytes. Atrial myocytes: WT: 133 cells, 4 mice; 4SA-Rad: 75 cells, 4 mice. **h**, Isoproterenol-induced fold-change in Ca^2+^ transient amplitude. Data are the mean ± s.e.m. ****P < 0.0001 by unpaired, two-tailed Student’s *t*-test (*n* = 133, 75 atrial cardiomyocytes from 4 and 4 mice, from left to right). **i**, Amplitude of basal Ca^2+^ transient for ventricular and atrial cardiomyocytes. Data are mean ± s.e.m. Unpaired, two-tailed Student’s *t*-test. ****P < 0.0001; *P < 0.05 (*n* = 462, 507, 133 and 75 cardiomyocytes from 8, 10, 4 and 4 mice, respectively, from left to right).

How relevant is the modulation of Ca^2+^ channels in the broader context of changes in the Ca^2+^ transient and contractility, processes that involve not only the Ca_V_1.2 channel, but many other ion channels and transporters, all targets of adrenergic signaling? Excitation–contraction coupling in myocytes is initiated by myocyte membrane depolarization, leading to the opening of Ca_V_1.2 channels and the influx of extracellular Ca^2+^ that in turn activates RyR2, the SR Ca^2+^ release channels (Fig. 3a). The transient increase in cytosolic Ca^2+^ concentration induces contraction. Electrical field-stimulation-induced Ca^2+^ transients of WT and 4SA-Rad ventricular cardiomyocytes, detected by the ratiometric Ca^2+^ indicator Fura2-AM, were quantified before and after a 2-min superfusion of vehicle, isoproterenol or forskolin (Fig. 3b). Thereafter, the Ca^2+^ content of the SR was assessed by rapid infusion of caffeine, which induces release of SR Ca^2+^.

As expected, isoproterenol and forskolin, but not vehicle, increased the Ca^2+^ transient amplitude in WT ventricular cardiomyocytes (Fig. 3c–f). The effect of isoproterenol on the Ca^2+^ transient amplitude was even more profound in WT atrial cardiomyocytes (Fig. 3g,h). The extent of isoproterenol- and forskolin-induced change in the Ca^2+^ transient of WT ventricular cardiomyocytes was inversely related to the basal transient amplitude (Fig. 3d,e), similar to the inverse relationship between basal Ca^2+^ current and the extent of the response to the β-adrenergic agonist^25^.

Compared with WT ventricular myocytes, the increase in the Ca^2+^ transient by adrenergic agonists was markedly attenuated in 4SA-Rad ventricular myocytes by 70% (80% increase in 4SA-Rad versus 260% increase in WT) (Fig. 3d–f). Compared with WT atrial myocytes, the isoproterenol-induced increase in Ca^2+^ transient was attenuated by 93% (32% increase in 4SA-Rad versus 490% increase in WT) in 4SA-Rad atrial myocytes (Fig. 3g,h). The 4SA-Rad cardiomyocytes exhibited increased amplitude of the basal Ca^2+^ transient compared with WT cardiomyocytes (Fig. 3i), despite the modest reduction in basal Ca^2+^ conductance (Fig. 1d). This implies at least some downstream compensatory signaling to attenuate the loss of adrenergic augmentation of the Ca^2+^ transient amplitude.

**Fig. 4 |:**
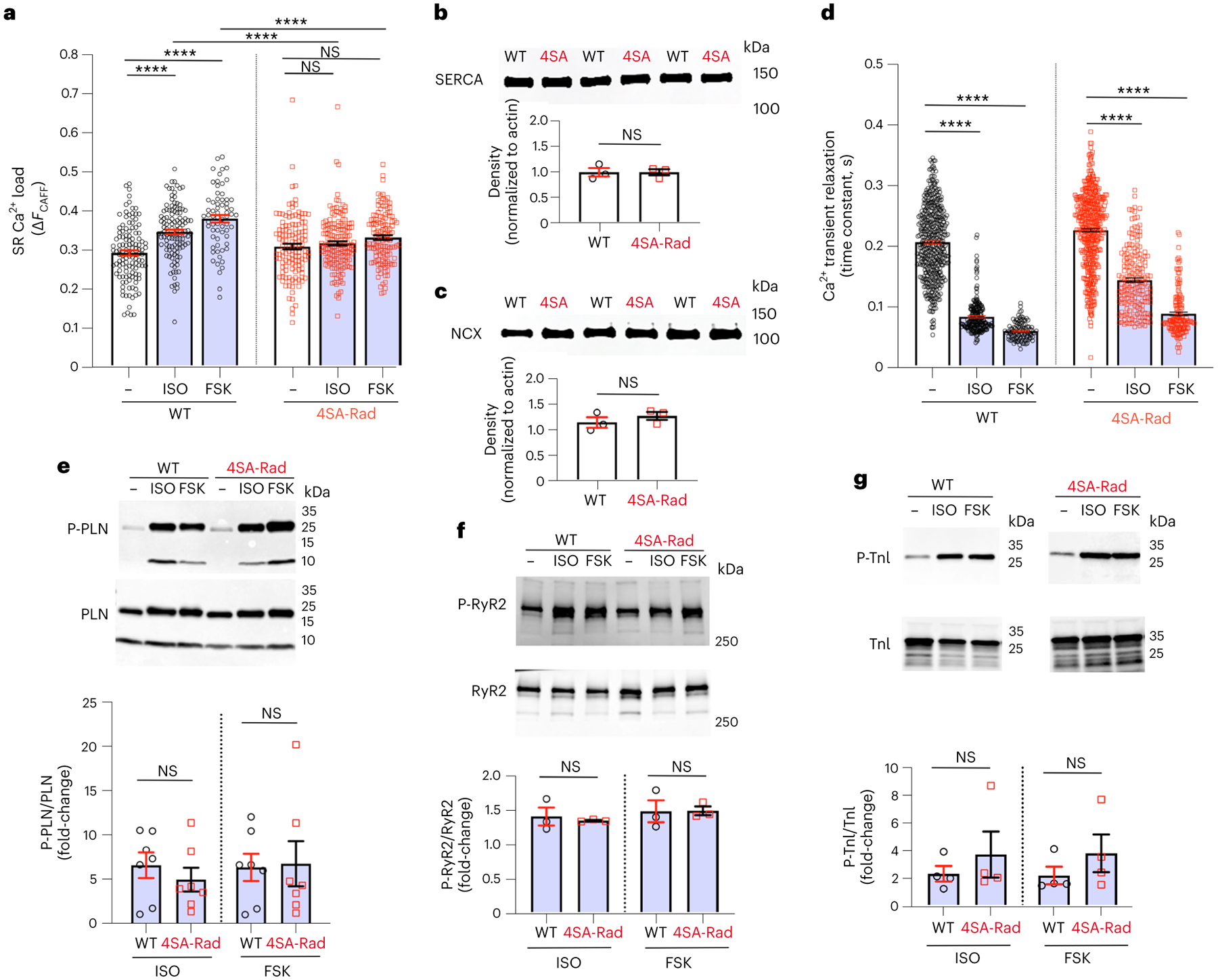
Adrenergic signaling pathways are fully functional in 4SA-Rad mice. **a**, Graph of amplitude of Ca^2+^ transient after caffeine in control, isoproterenoland forskolin-treated cardiomyocytes. Data are the mean ± s.e.m. *P* < 0.0001 by one-way ANOVA; ****P < 0.0001, **P < 0.01 by Sidak’s multiple-comparison test (*n* = 121, 120, 66, 129, 165 and 129 cardiomyocytes from 3, 4, 5, 3, 4 and 5 mice, respectively). **b**, Anti-SR Ca^2+^-ATPase (SERCA) antibody western blot of protein lysates of cardiomyocytes isolated from WT and 4SA-Rad mice. Lower: graph of density normalized to β-actin. Data are the mean ± s.e.m. Unpaired, two-tailed Student’s *t*-test. *P* > 0.99 (*n* = 3 mice in each group). **c**, Same as **b** except with anti-NCX antibody western blot. Lower: graph of density normalized to β-actin. Data are the mean ± s.e.m. Unpaired, two-tailed Student’s *t*-test *P* = 0.38 (*n* = 3 mice in each group). **d**, Graph of time constant of Ca^2+^ transient relaxation under basal conditions and after either isoproterenol or forskolin. Data are the mean ± s.e.m. *P* < 0.0001 by one-way ANOVA, ****P < 0.0001 by Tukey’s multiple-comparison test (*n* = 462, 225, 109, 478, 183, 149 cardiomyocytes from 12, 4, 3, 13, 5 and 5 mice, respectively, from left to right). **e**, Anti-phospho-PLN (P-PLN) antibody (upper) and anti-PLN antibody (middle) western blots of protein lysates of cardiomyocytes. Lower: graph of P-PLN density normalized to PLN density. Data are the mean ± s.e.m. *P* = 0.43 and *P* = 0.89, from left to right, by unpaired, two-tailed Student’s *t*-test (*n* = 7 mice in each group). **f**, Same as in **e** except with anti-phospho-S2808 RyR2 antibody (upper) and anti-RyR2 antibody (middle) western blots. Lower: graph of P-RyR2 density normalized to RyR2 density. Data are the mean ± s.e.m. *P* = 0.66 and *P* = 0.97, from left to right, by unpaired, two-tailed Student’s *t*-test (*n* = 3 mice in each group). **g**, Same as in **e** except with anti-S23/S24 TnI antibody (upper) and anti-TnI antibody (middle) western blots. Lower: graph of P-TnI density normalized to TnI density. Data are mean ± s.e.m. *P* = 0.46 and *P* = 0.33, from left to right, by unpaired, two-tailed Student’s *t*-test (*n* = 4 mice in each group).

Assessed by the magnitude of caffeine-induced SR Ca^2+^ release, isoproterenol and forskolin increased SR Ca^2+^ load in WT but not 4SA-Rad cardiomyocytes (Fig. 4a), despite equivalent expression of SERCA and the Na^+^/Ca^2+^ exchanger (NCX) (Fig. 4b,c). Although adrenergic augmentation of the Ca^2+^ transient was markedly diminished in the 4SA-Rad cardiomyocytes, other fundamental adrenergic signaling pathways remained fully functional in these mice. First, both isoproterenol and forskolin accelerated the rate of Ca^2+^ reuptake (Fig. 4d). Second, isoproterenol and forskolin induced phosphorylation of PLN (Fig. 4e), RyR2 (Fig. 4f) and troponin I (TnI) (Fig. 4g) in ventricular myocytes isolated from both WT and 4SA-Rad mice. Taken together, the bulk of the adrenergic Ca^2+^ transient and contraction augmentation in isolated cardiomyocytes depends on Rad phosphorylation and the subsequent increase in Ca^2+^ influx.

### Rad phosphorylation is required for increased contractility

**Extended Data Fig. 2 |:**
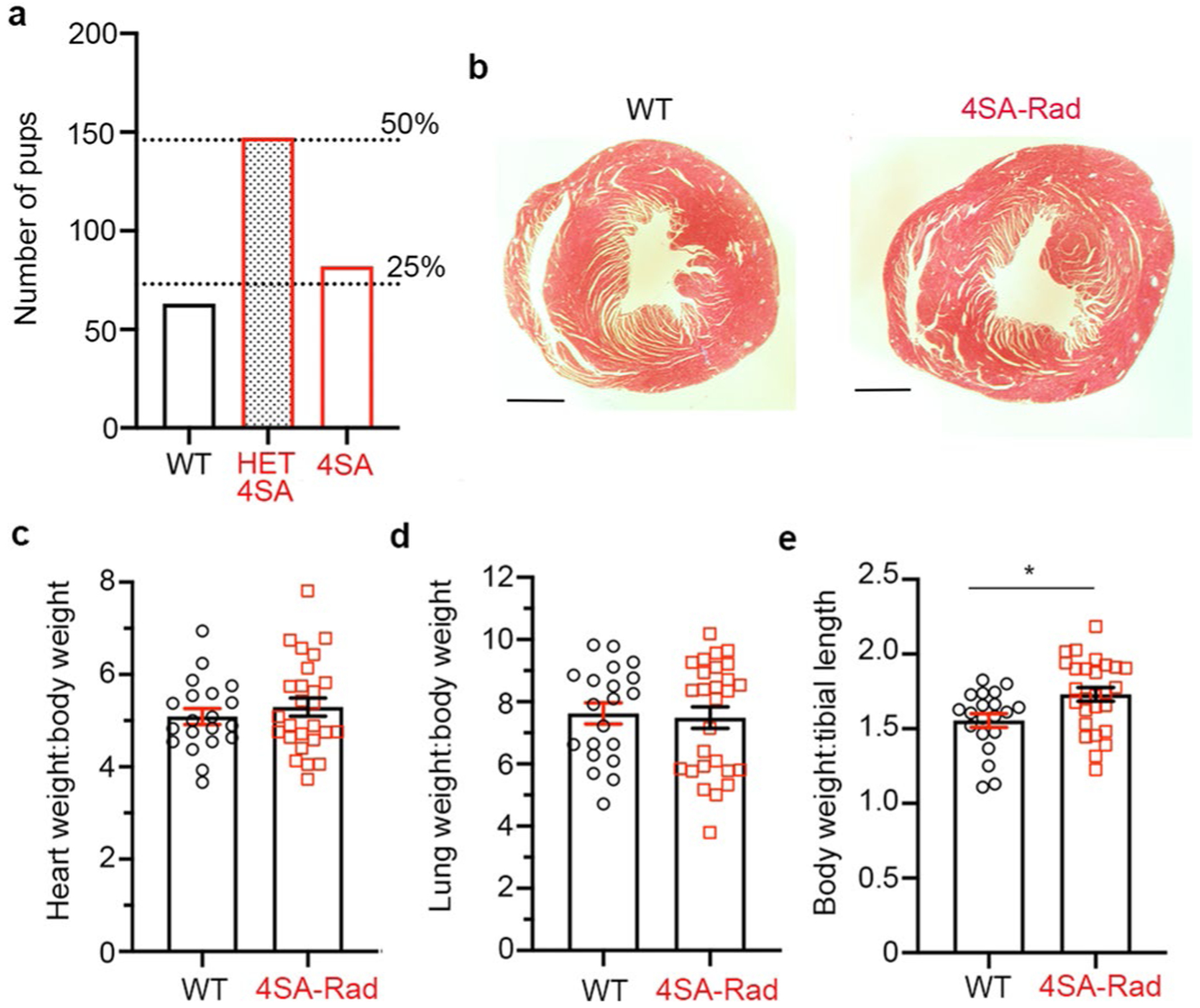
Characterization of 4SA-Rad knock-in mice. **a**, Graph of number of 292 pups from 43 litters of heterozygous 4SA-Rad mice crosses. Dashed lines are the expected number of pups representing 25% (WT and 4SA-Rad) and 50% (heterozygous 4SA-Rad). *P* = 0.2885 observed vs. expected by Chi-square. The discrepancy between observed and expected is not significant. **b**, Trichrome stain of cross-sections of WT and 4SA-Rad heart from 5-month-old mice. Representative of 15 WT mice and 24 4SA-Rad mice. **c**, Graph of heart weight normalized to body weight. Data are mean ± s.e.m.; *P* = 0.45 by unpaired two-tailed *t*-test. N = 20 WT mice and 26 4SA-Rad mice. **d**, Graph of lung weight normalized to body weight. Data are mean ± s.e.m.; *P* = 0.79 by unpaired two-tailed *t*-test. N = 20 WT mice and 27 4SA-Rad mice. **e**, Graph of body weight normalized to tibial length of mice. Data are mean ± s.e.m.; * *P* < 0.05 by unpaired two-tailed *t*-test. N = 20 WT mice and 27 4SA-Rad mice.

Mice expressing 4SA-Rad were born at normal Mendelian ratios (Extended Data Fig. 2a) and their survival was equivalent to WT animals up to age at least 6 months. Histological examination of 4SA-Rad hearts did not display increased fibrosis or changes in wall thickness (Extended Data Fig. 2b). Heart and lung weights in WT and 4SA-Rad mice did not differ (Extended Data Fig. 2c,d). Body weight of 4SA-Rad mice was slightly increased compared with littermate WT mice (Extended Data Fig. 2e).

**Extended Data Fig. 3 |:**
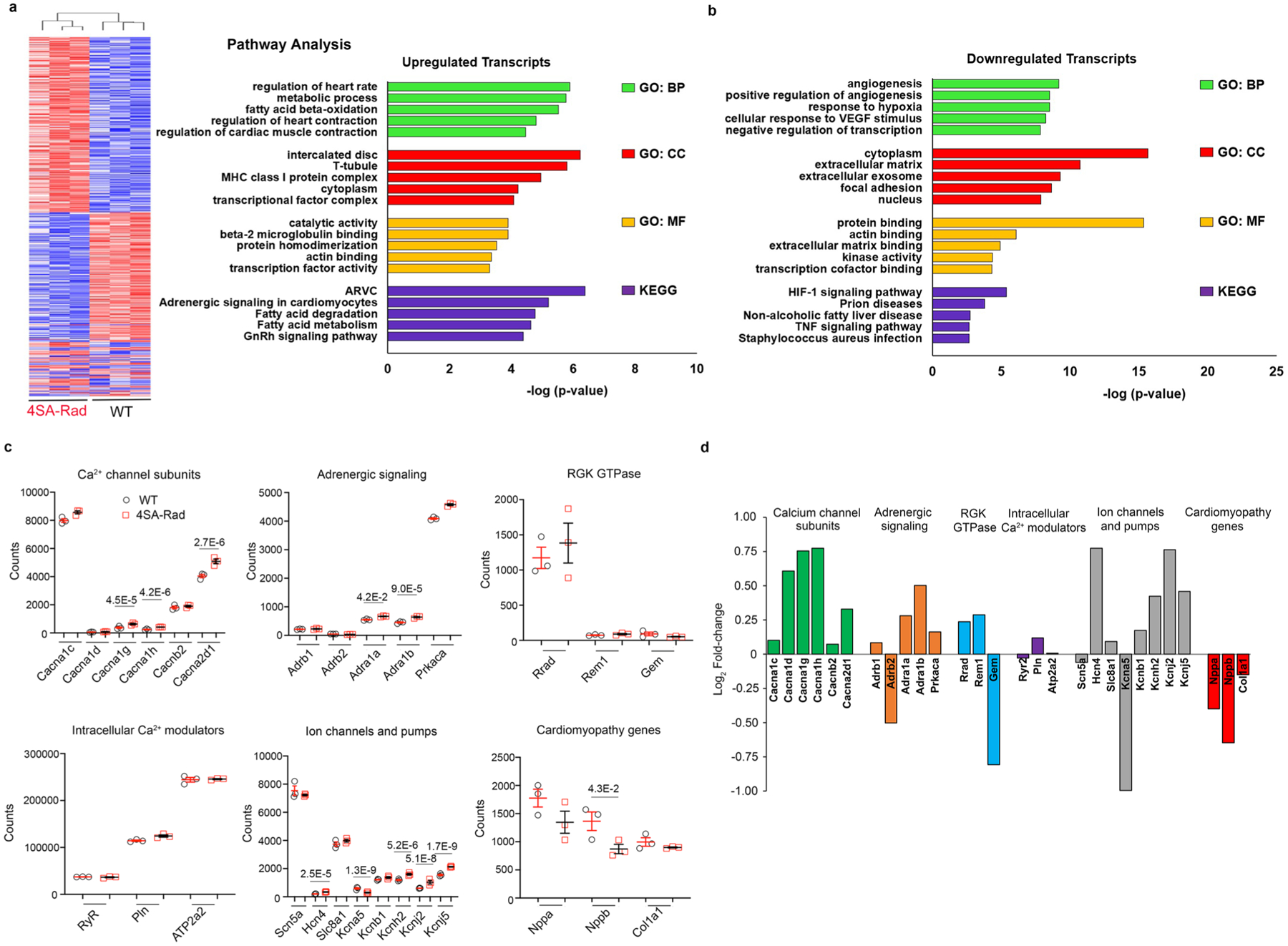
Myocardial transcriptional profiling of 4SA-Rad knock-in mouse model. **a**, Hierarchical clustering of 3 homozygous 4SA-Rad and 3 WT mice based on significantly dysregulated transcripts. **b**, Gene ontology and KEGG pathway analysis of significantly upregulated and downregulated transcripts. **c**, Graphs of normalized counts for selected cardiac genes. *P*_adjusted_ shown in figure for those transcripts that are significantly changed between 4SA-Rad and WT mice (see Methods and Supplementary Table 2). **d**, Fold-change of transcripts in 4SA-Rad:WT mice.

Bulk RNA-seq demonstrated modest changes with 607 genes downregulated and 445 genes upregulated in homozygous 4SA-Rad hearts compared with WT hearts (Extended Data Fig. 3a,b). Upregulated *Kyoto Encyclopedia of Genes and Genomes* (KEGG) pathways in the 4SA-Rad hearts included arrhythmogenic right ventricular cardiomyopathy, adrenergic signaling, regulation of heart contraction and metabolism (Extended Data Fig. 3b). Notably, the transcript levels for Ca_V_1.2 α_1C_- and β_2B_-subunits, RyR2, SERCA2, PLN and Rad and other RGK GTPase family members were not substantially altered (Extended Data Fig. 3c,d and Supplementary Table 2). In contrast, transcripts levels for adrenergic receptors (*Adra1a*, *Adra1b*), HCN4, Ca_V_3.1 (*Cacn1g*) and Ca_V_3.2 (*Cacn1h*), Ca_V_ α_2_δ_1_ (*Cacna2d1*) and several K^+^ channels were upregulated (Extended Data Fig. 3c,d). We surmise that these changes reflect compensation for the loss of adrenergic regulation of Ca^2+^ channels.

**Fig. 5 |:**
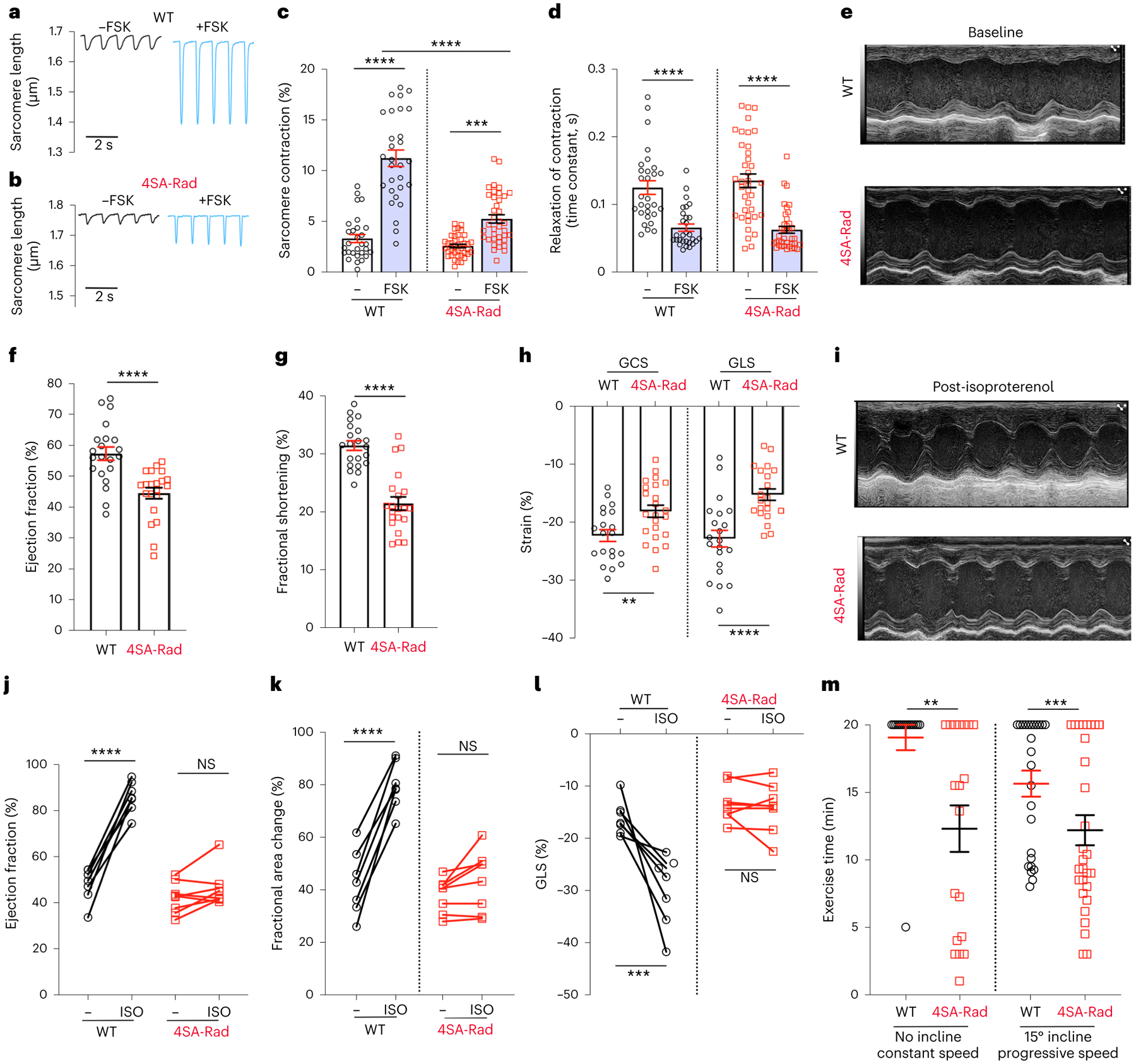
Phosphorylation of Rad required for regulation of contractility. **a**,**b**, Pacing-induced change in sarcomere length before and after superfusion of forskolin. **c**, Graph showing field-stimulation-induced percentage change in sarcomere length. Data are the mean ± s.e.m. *P* < 0.0001 by one-way ANOVA; ***P < 0.001, ****P < 0.0001 by Sidak’s multiple-comparison test (*n* = 29, 29, 38 and 38 cardiomyocytes from 9, 9, 8 and 8 mice, respectively, from left to right). **d**, Graph of single-exponential decay time constant of reduction in contraction. Data are the mean ± s.e.m. ****P < 0.0001 by unpaired, two-tailed Student’s *t*-test (*n* = 29, 29, 38 and 38 cardiomyocytes from 9, 9, 8 and 8 mice, respectively, from left to right). **e**,**i**, Representative M-mode echocardiographic recordings before (**e**) and after (**i**) intraperitoneal injection of isoproterenol (ISO). **f**,**g**, Graphs of ejection fraction and fractional shortening from echocardiograms. Data are the mean ± s.e.m. ****P < 0.0001 by unpaired, two-tailed Student’s *t*-test (*n* = 21 mice for each group). **h**, Graphs of GCS and GLS. Data are the mean ± s.e.m. **P < 0.01, ****P < 0.0001 by unpaired, two-tailed Student’s *t*-test (*n* = 20, 23, 20 and 21 mice, from left to right). **j**, Graph of ejection fraction before and after isoproterenol. Data are the mean ± s.e.m. ****P < 0.0001 and *P* = 0.29 by unpaired, two-tailed Student’s *t*-test (*n* = 7 and 8 mice, from left to right). **k**, Graph of fractional area of change before and after isoproterenol. Data are the mean ± s.e.m. ****P < 0.0001 and *P* = 0.27 by unpaired, two-tailed Student’s *t*-test (*n* = 7 and 8 mice, from left to right). **l**, Graph of GLS before and after isoproterenol. ***P < 0.001 and *P* = 0.69 by unpaired, two-tailed Student’s *t*-test (*n* = 7 and 8 mice, from left to right). **m**, Graph of exercise time for WT and 4SA-Rad mice using either no incline or 15° incline with progressively increasing speed. Data are mean ± s.e.m. *P < 0.05, ***P < 0.001 by Mann–Whitney *U*-test (*n* = 16 mice (7 male/9 female), 19 mice (10 male/9 female), 25 mice (12 male/13 female) and 29 mice (15 male/14 female), from left to right).

To assess the role of Rad phosphorylation in cardiac contractility in vitro, we measured the changes in pacing-induced sarcomere length before and after exposure to forskolin. In WT cardiomyocytes, the sarcomere contraction increased from 3.4% to 11.3% (absolute difference 7.9%) in response to forskolin (Fig. 5a,c). In contrast, the sarcomere contraction increased only from 2.7% to 5.3% (absolute difference 2.6%) in forskolin-treated 4SA-Rad ventricular myocytes (Fig. 5b,c). The forskolin-induced acceleration in the relaxation speed after the pacing-induced contraction was equivalent in WT and 4SA-Rad cardiomyocytes (Fig. 5d), consistent with the normal adrenergic signaling to PLN and TnI in 4SA-Rad cardiomyocytes (Fig. 4e,g).

**Extended Data Fig. 4 |:**
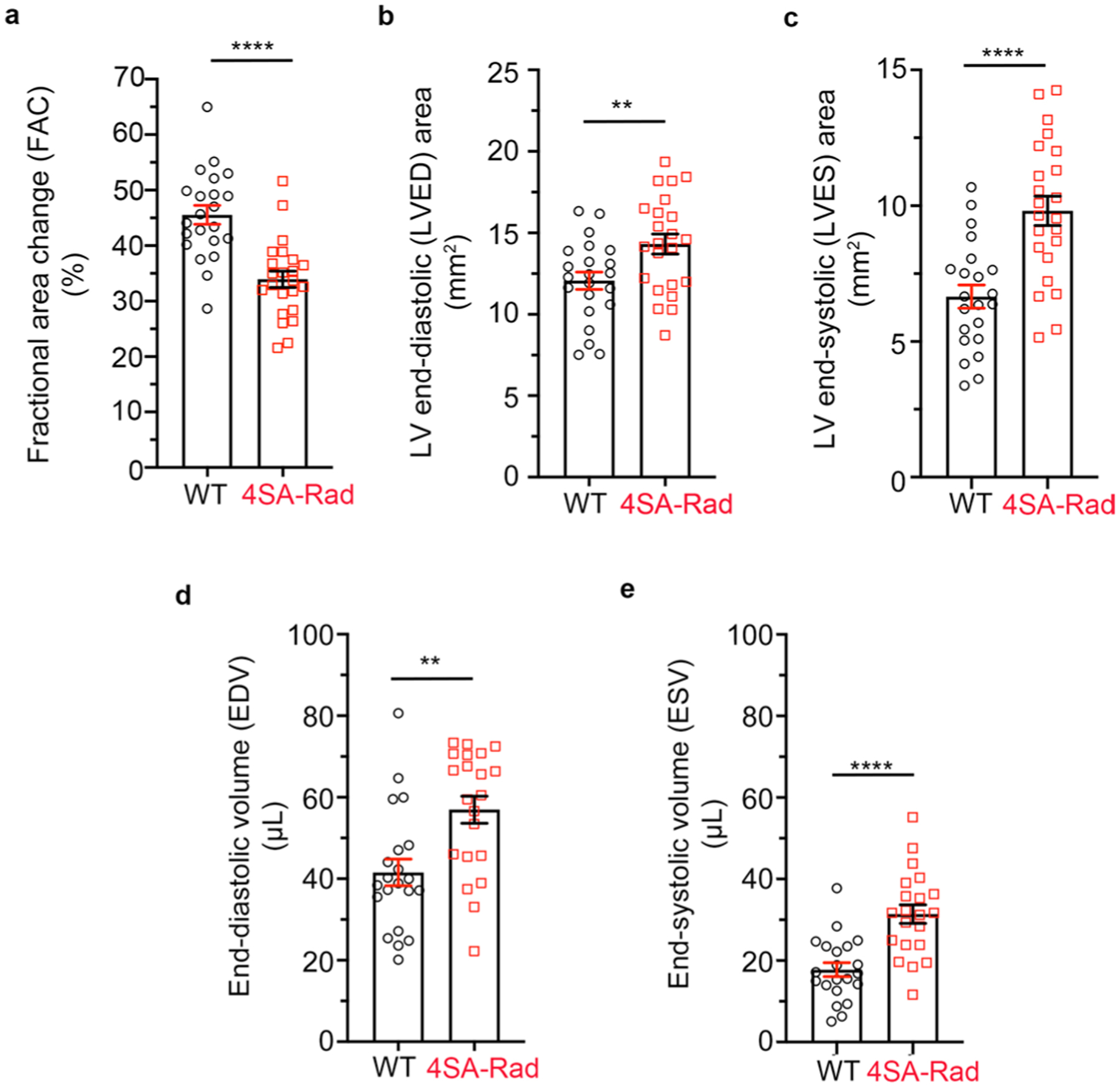
Echocardiographic characteristics of WT and 4SA-Rad mice. **a**, Graph of basal fractional area of change (FAC) of WT and 4SA-Rad mutant mice. Data are mean ± s.e.m.; ****P < 0.0001 by unpaired two-tailed *t*-test. N = 23 WT mice and 22 4SA-Rad mice. **b**, Graph of basal left ventricular (LV) end-diastolic (LVED) area. Data are mean ± s.e.m.; **P < 0.01 by unpaired two-tailed *t*-test. N = 22 WT mice and 23 4SA-Rad mice. **c**, Graph of LV end-systolic (LVES) area. Data are mean ± s.e.m.; ****P < 0.0001 by unpaired two-tailed *t*-test. N = 22 WT mice and 23 4SA-Rad mice. **d**, Graph of end-diastolic volume (EDV). Data are mean ± s.e.m.; **P < 0.01 by unpaired two-tailed *t*-test. N = 21 WT mice and 21 4SA-Rad mice. **e**, Graph of end-systolic volume (ESV). Data are mean **±** s.e.m.; ****P < 0.0001 by unpaired two-tailed *t*-test. N = 21 WT mice and 21 4SA-Rad mice.

To assess the role of Rad phosphorylation in cardiac contractility in vivo, we performed echocardiography on isoflurane-anesthetized WT and 4SA-Rad mice. In the 4SA-Rad mice, we found ~25% reduction in baseline contractility, with expansion of both end-diastolic and end-systolic volumes of the left ventricle, and a reduction in both global circumferential strain (GCS) and global longitudinal strain (GLS; Fig. 5e–h and Extended Data Fig. 4a–e). Next, we assessed the effects of intraperitoneal injection of isoproterenol on cardiac contractility (Fig. 5i). Isoproterenol increased the ejection fraction and fractional area of change by 81% and 86% (absolute difference 38% and 37%), respectively, in WT mice, but increased the ejection fraction and fractional area of change by only 9% and 14% (absolute difference 3% and 5%), respectively, in 4SA-Rad mice (Fig. 5j,k). Speckle tracking-based strain analysis confirmed the pronounced attenuation of the isoproterenol effect in 4SA-Rad mice (Fig. 5l). These data demonstrate that adrenergic augmentation of cardiac contractility in vivo strongly depends on Rad phosphorylation and the subsequent increased Ca^2+^ influx.

We subjected WT and 4SA-Rad mice to treadmill exercise testing. After 2 d of treadmill acclimation and training, mice were subjected to a 20-min exercise session at an incline of either 0° or 15°. At constant speed (25 cm s^−1^) and no incline, substantially more 4SA-Rad mice than WT mice failed the 20-min testing session, with a shorter latency to failure in the 4SA-Rad mice (Fig. 5m). Although the number of mice failing to complete the 20-min testing session increased for both WT and 4SA-Rad groups at an incline of 15° and progressively increasing speed, the 4SA-Rad mice group was more prone to failure with reduced latency (Fig. 5m). Theoretically, we cannot exclude that phosphorylated Rad has roles in metabolism^26^ or in other tissues relevant to exercise capacity, such as skeletal muscle^27^. Still, exercise intolerance is a hallmark of heart failure and/or inability to increase cardiac output, and these findings are consistent with the expected consequences of diminished fight-or-flight responses.

### Adrenergic regulation of heart rate

In addition to increased myocyte contractility rate, the fight-or-flight response depends on increased heart rate. Cardiac automaticity is chiefly driven by membrane potential depolarization of sinoatrial nodal (SAN) cells during diastole, determined by the complex coupling of the ‘membrane clock’ and the ‘Ca^2+^ clock’^28^. The coupled clock model postulates that, along with the inward cation current through hyperpolarization-activated cyclic nucleotide-gated (HCN4) channels, spontaneous local Ca^2+^ release events and NCX depolarize the membrane causing activation of T-type and L-type Ca^2+^ channels (Ca_V_1.2 and Ca_V_1.3) and generation of the action potential^29^. Adrenergic agonists, via cAMP generation and PKA activation, accelerate pacemaker activity via integrated actions on multiple targets including HCN4, Ca^2+^ channels, RyR2, PLN/SERCA, NCX and K^+^ channels^29,30^. Ca_V_1.3 channels are critical for the initiation of pacemaker activity in dormant mouse SAN cells by β-adrenergic stimulation^31^. Rad is expressed in SAN cells and Rad knockout mice demonstrate increased intrinsic and sleep-phase heart rates consistent with a role of Rad in modulating Ca^2+^ current in SAN cells^32^. Using the 4SA-Rad mice generated in the present study, we assessed the role of adrenergic stimulation of Ca_V_1.2 and Ca_V_1.3 channels on sinus node function.

**Fig. 6 |:**
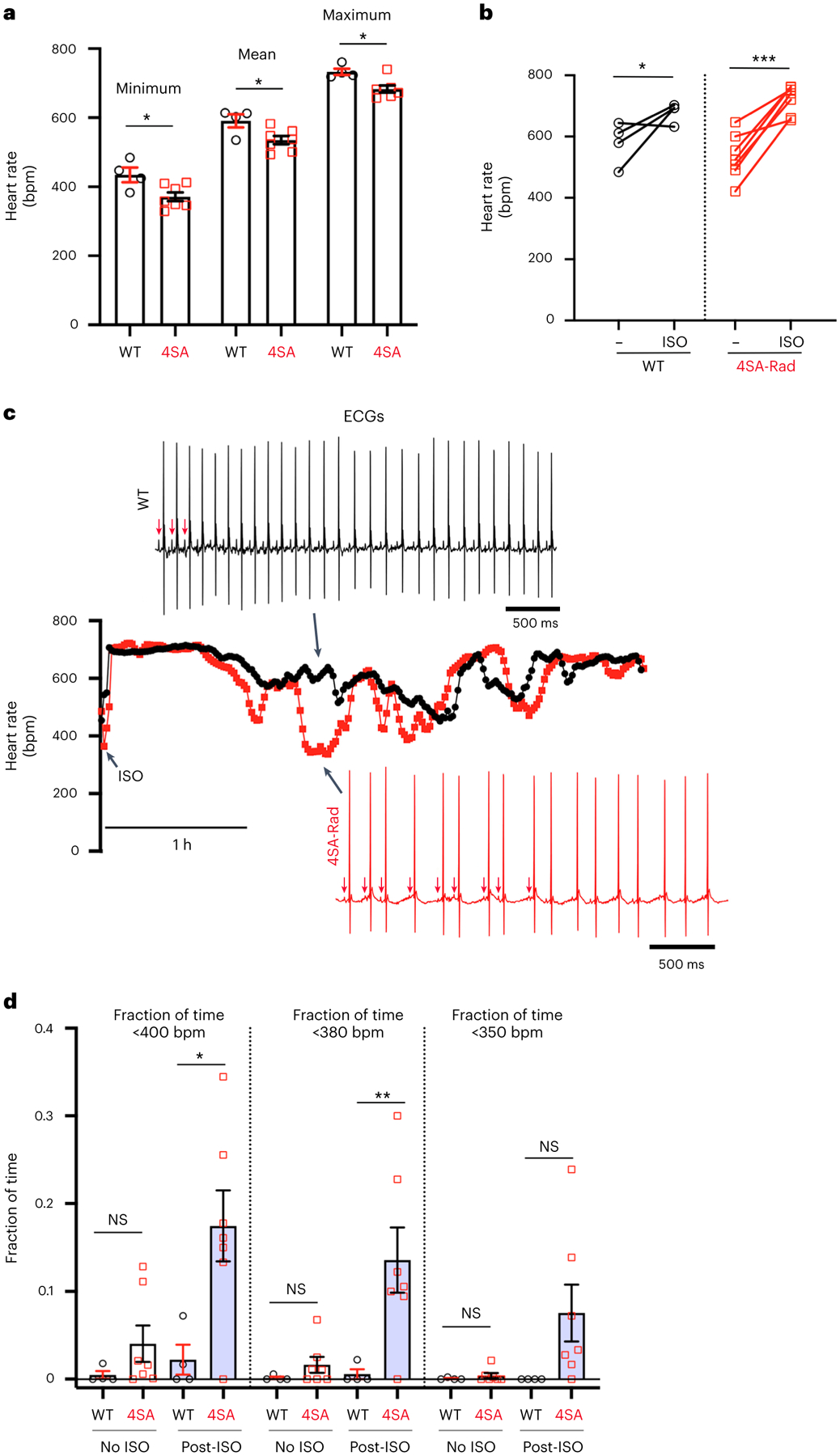
Phosphorylation of Rad required for regulation of heart rate in vivo. **a**, Graph of minimum, mean and maximum heart rate over a 24-h period. Data are the mean ± s.e.m. *P < 0.05 by unpaired, two-tailed Student’s *t*-test (*n* = 4 WT mice; 7 4SA-Rad mice). **b**, Graph of heart rate before and 10 min after intraperitoneal injection of isoproterenol. Data are the mean ± s.e.m. *P < 0.05, ***P < 0.001 by unpaired, two-tailed Student’s *t*-test. **c**, Representative diary plots of heart rate during 4-h period after intraperitoneal injection of isoproterenol. The black trace is a WT mouse and the red trace a 4SA-Rad mouse. Representative ECGs are shown for the indicated timepoint. The arrows point to atrial activations (P waves). **d**, Graph of fraction of time in which heart rates were <400 beats min^−1^ (bpm), 380 bpm or 350 bpm during a 24-h period without isoproterenol and a 3-h period post-isoproterenol injection. Data are the mean ± s.e.m. *P < 0.05, **P < 0.01 by unpaired, two-tailed Student’s *t*-test. Specific *P* values can be found in the associated Source data (see Supplementary Information) (*n* = 4 WT and 7 4SA-Rad mice).

We implanted radio-telemeters in mice and recorded electrocardiograms (ECGs) under basal conditions to assess sinus node function. The 4SA-Rad mice demonstrated a reduction in minimum, mean and maximum heart rate over a 24-h period (Fig. 6a). Acute injection of isoproterenol induced an increase in maximum heart rate in both WT and 4SA-Rad mice (Fig. 6b). During the subsequent several hours after isoproterenol injection, however, we observed a greater slowing of the heart rate in 4SA-Rad mice, marked by prolonged episodes of irregularity and slow heart rates below 400 beats min^−1^, which was rarely observed in WT mice (Fig. 6c,d). These data suggest that Rad phosphorylation has important protective effects in preventing slow heart rates and stabilizing sinus node pacemaker activity during periods of high stress. As heart rate increases with isoproterenol, the reduction in exercise capacity is not related to a lack of chronotropic response.

### Rad-inhibited channels required for adrenergic regulation

**Extended Data Fig. 5 |:**
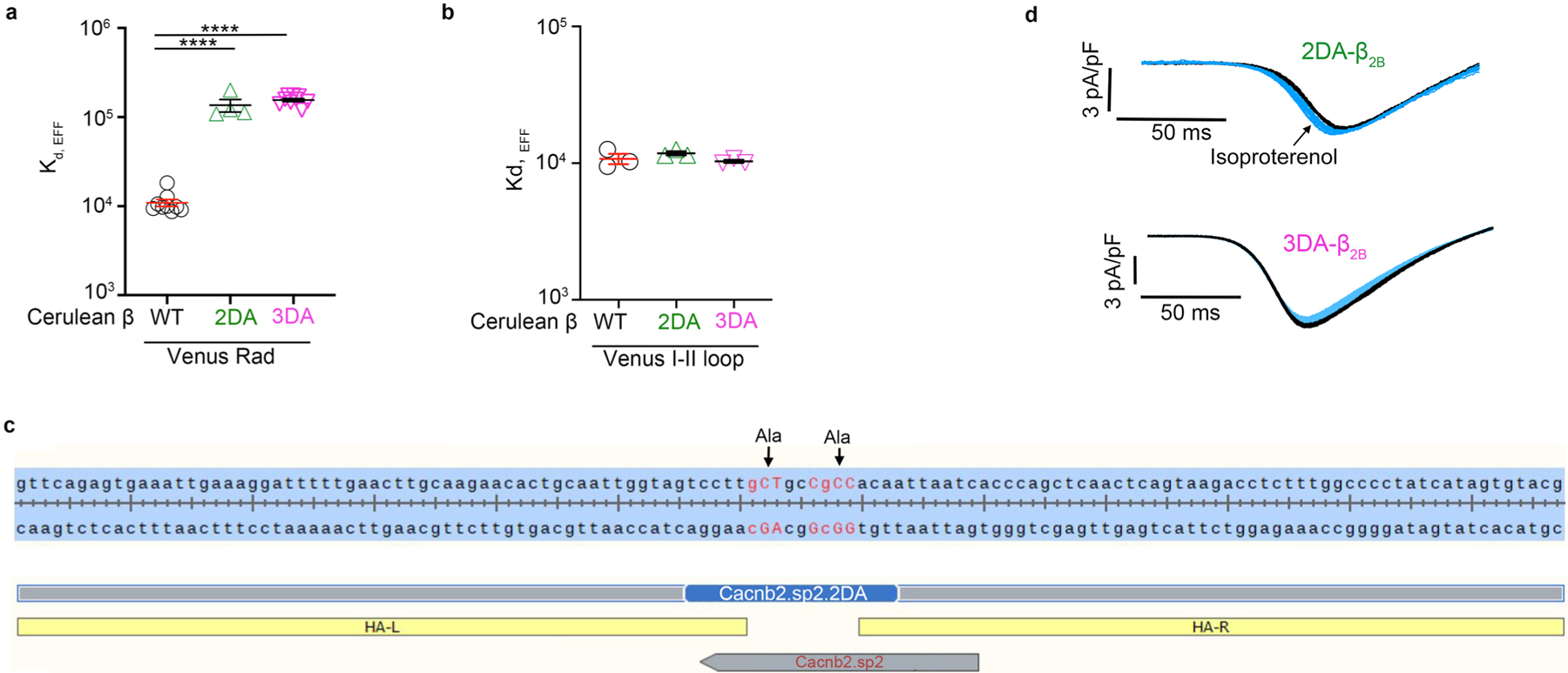
Disrupting β and Rad interaction by mutation of β_2B_. **a**, FRET two-hybrid binding isotherms were determined for N-terminal Cer-tagged WT β_2B_, 2DA-β_2B_ and 3DA-β_2B_, and N-terminal Ven-tagged WT Rad. Graph summarizing Kd,EFF for WT, 2DA-β_2B_ and 3DA-β_2B_. Data are mean ± s.e.m.; **** *P* < 0.0001 by unpaired two-tailed *t*-test. n = 9, 4 and 4 independent replicates/experiments, from left to right. **b**, FRET two-hybrid binding isotherms were determined for N-terminal Cer-tagged WT β_2B_, 2DA-β_2B_ and 3DA-β_2B_, and N-terminal Ven-tagged I-II loop of α_1C_. Graph summarizing Kd,EFF for WT, 2DA-β_2B_ and 3DA-β_2B_. Data are mean ± s.e.m.; *P* = 0.36 and P = 0.66 by unpaired two-tailed *t*-test. n = 3 independent replicates/experiments for all groups. **c**, Schematic depicting approach for creation of 2DA-β_2B_ knock-in mouse. Guides and single-strand oligodeoxynucleotide as the donor template with Ala-substitutions for the two Asp residues in exon 11 were tested by Genome Engineering and iPSC Center (GEiC) at the Washington University in St. Louis. HA-L = homology arm-left, HA-R = homology arm-right. **d**, Ba^2+^ current elicited by voltage ramp every 6 s, with black traces obtained before and blue traces obtained after ISO in 2DA-β_2B_ and 3DA-β_2B_ ventricular myocytes.

**Fig. 7 |:**
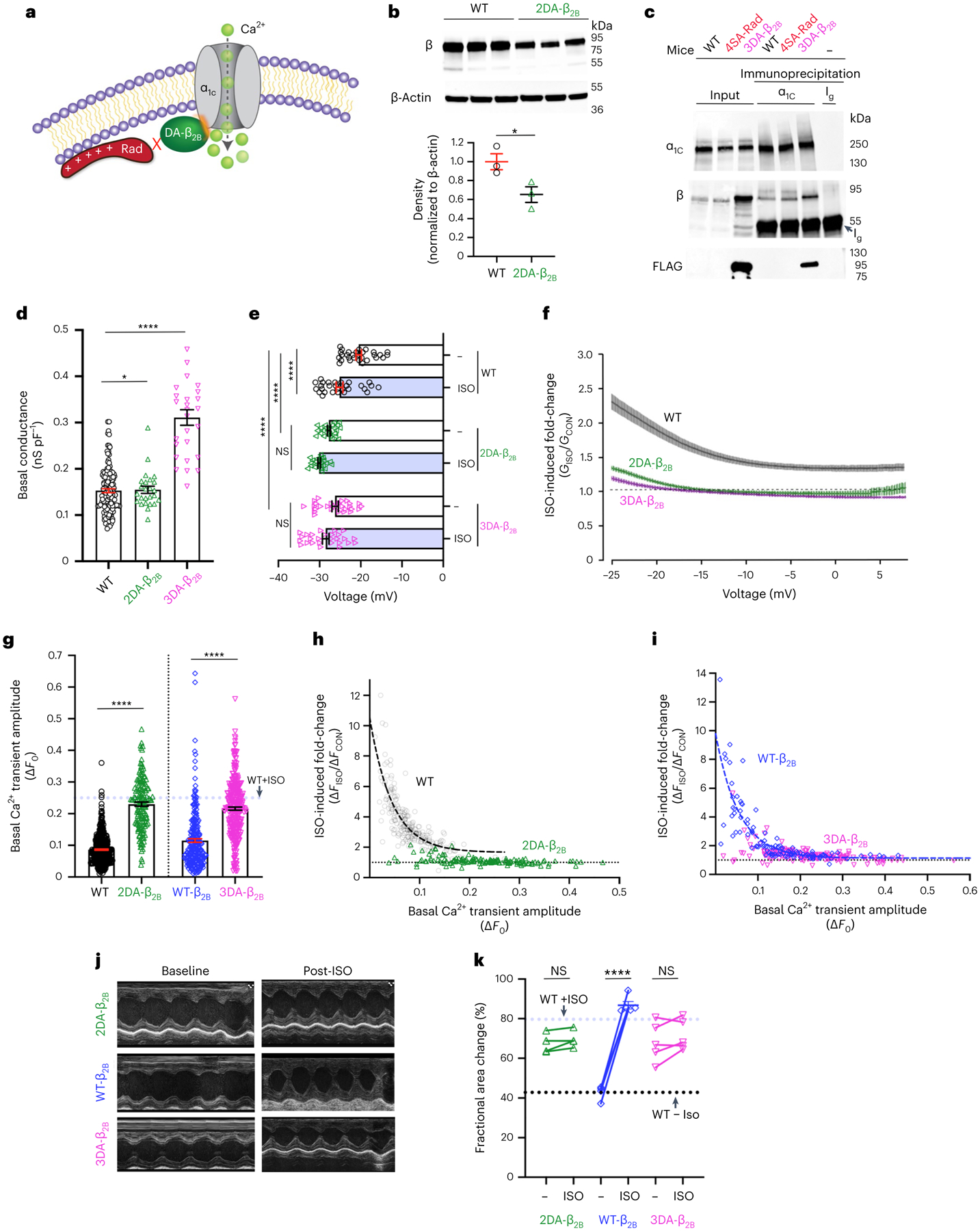
Augmentation of contractility requires Rad-bound Ca^2+^ channels under basal conditions. **a**, Schematic depicting mutation of the Ca_V_β subunit (DA-β) that prevents Rad binding. **b**, Anti-β subunit antibody (upper) and anti-β-actin (middle) antibody western blots. Lower: graph of densitometry, normalized to β-actin. Data are the mean ± s.e.m. *P < 0.05 two-tailed, unpaired Student’s *t*-test (*n* = 3 mice in each group). **c**, Anti-α_1C_, anti-β and anti-FLAG antibody western blots of anti-α_1C_ immunoprecipitation, representing three similar experiments. Ig, heavy chain of immunoglobulin. **d**, Graph of basal conductance density at −20 mV acquired using a ramp protocol. Data are the mean ± s.e.m. *P < 0.05; ****P < 0.0001 two-tailed, unpaired Student’s *t*-test (*n* = 28, 24 and 24 cardiomyocytes from 3, 4 and 3 mice, respectively, from left to right). **e**, Graph of *V*_50_. *P* < 0.0001 by one-way ANOVA; ****P < 0.0001 by Sidak’s multiple-comparison test. WT: 29, 29, 23, 23, 27 and 27 cells from 3, 3, 4, 4, 3 and 3 mice, respectively, from top to bottom. **f**, Ratio of Ba^2+^ currents after isoproterenol to before isoproterenol versus voltage. Data are the mean ± s.e.m. Sample size same as **e**. **g**, Graph of basal Ca^2+^ transient amplitude. The dashed blue line is the mean for WT + isoproterenol. The WT data are the same as in Fig. 3i. Data are the mean ± s.e.m. ****P < 0.0001 by unpaired, two-tailed Student’s *t*-test (*n* = 462, 151, 245, 316 cardiomyocytes from 8, 3, 3 and 6 mice, respectively, from left to right). **h**, Scatter plot of isoproterenol-induced fold-change of Ca^2+^ transient amplitude versus basal transient amplitude in WT (gray—data same as Fig. 3d) and 2DA-β_2B_ (green) myocytes. The dashed black line is a single-exponential fit of WT data (*n* = 225 WT cells, 4 mice; 151 2DA-β_2B_ cells, 3 mice). **i**, Scatter plot of isoproterenol-induced fold-change of Ca^2+^ transient amplitude versus basal transient amplitude. The blue dashed line is a single-exponential fit of transgenic WT β_2B_ data (*n* = 106 WT-β_2B_ cells, 3 mice; 148 3DA-β_2B_ cells, 3 mice). **j**, Representative M-mode recordings. **k**, Graph of fractional area of change. The dashed lines are means for WT mice without and with isoproterenol. Data are the mean ± s.e.m. ****P < 0.0001 by unpaired, two-tailed Student’s *t*-test (*n* = 5 mice from each group).

Expression of Rad profoundly inhibits the open probability of Ca_V_1.2 channels^15,33^. The presence of Ca^2+^ current in cardiomyocytes under basal conditions implies that a substantial fraction of Ca^2+^ channels is not bound to Rad, and that Rad-bound Ca^2+^ channels form the functional reserve of Ca^2+^ influx and cardiac contractility. Rad-null mice demonstrate high basal contractility and blunted adrenergic responses^34,35^. To test whether the Rad-bound Ca^2+^ channel form the contractile reserve, we generated mice in which the interaction between the Ca^2+^ channel β-subunit and Rad is reduced (Fig. 7a). Previous studies showed that substituting three aspartic acid β_2B_ residues, Asp244, Asp320 and Asp322, in the human β_2B_-subunit, with alanine (3DA) attenuated Rad binding to the Ca^2+^ channel β-subunit^36,37^, which we confirmed using a flow cytometry, Förster resonance energy transfer (FRET), two-hybrid assay in HEK293 cells^24^ (Extended Data Fig. 5a). To facilitate generation of knock-in mice, because the aspartate-coding residues in exon 9 and exon 11 are separated by 9.3 kb, we determined that alanine substitutions of only the two aspartate residues in exon 11 (2DA) are sufficient to reduce Rad–β_2B_-subunit interaction (Extended Data Fig. 5a). These mutations in the β-subunit did not affect the interaction between the I–II loop of α_1C_ and β, also assessed by a FRET assay (Extended Data Fig. 5b).

We introduced, via clustered regularly interspaced short palindromic repeats (CRISPR)–Cas9 gene editing, alanine substitutions of these two aspartate residues in exon 11 of the endogenous murine *Cacnb2* locus (2DA-β_2B_ mice) (Extended Data Fig. 5c). Unexpectedly, expression of 2DA-β_2B_ was reduced by 35% in the knock-in mice compared with the β_2B_-subunit in the WT mice (Fig. 7b), which could confound interpretation of adrenergic stimulation^14^. To circumvent the reduced expression of the mutant β_2B_-subunit, we also created transgenic mice with cardiomyocyte-specific expression of 3× FLAG-epitope-tagged-3DA-β_2B_ proteins (3DA-β_2B_ mice). Transgenic overexpression of the WT β_2B_-subunit does not prevent adrenergic stimulation of Ca_V_1.2 current^14,15^. The 3× FLAG-epitope-tagged 3DA-β_2B_ proteins replace endogenous β-subunits in the Ca_V_1.2 complex, assessed by immunoprecipitation of the α_1C_-subunit and western blotting for β_2B_ and 3× FLAG-3DA-β_2B_ (Fig. 7c).

In ventricular cardiomyocytes isolated from mice with mutant Ca^2+^ channel β subunits that cannot bind Rad, the basal conductance was increased (Fig. 7d) and the *V*_50_ for activation was substantially hyperpolarized; neither *V*_50_ nor conductance was substantially changed by adrenergic agonists (Fig. 7e,f and Extended Data Fig. 5d). Similarly, the amplitude of the Ca^2+^ transient was increased under basal conditions to almost the levels of isoproterenol-treated WT cardiomyocytes (Fig. 7g) and did not substantially change after isoproterenol administration in 2DA-β_2B_ or 3DA-β_2B_ cardiomyocytes, in contrast to WT or transgenic WT β_2B_ cardiomyocytes (Fig. 7h,i). Basal cardiac contractility, assessed by echocardiography, was increased and the augmentation of contractility by isoproterenol was blunted in both lines of mice compared with either WT or WT β_2B_-expressing transgenic mice controls (Fig. 7j,k). These findings reveal that a subpopulation of Rad-bound Ca^2+^ channels is required for sympathetic nervous system regulation of Ca^2+^ influx, transient and contractility.

**Fig. 8 |:**
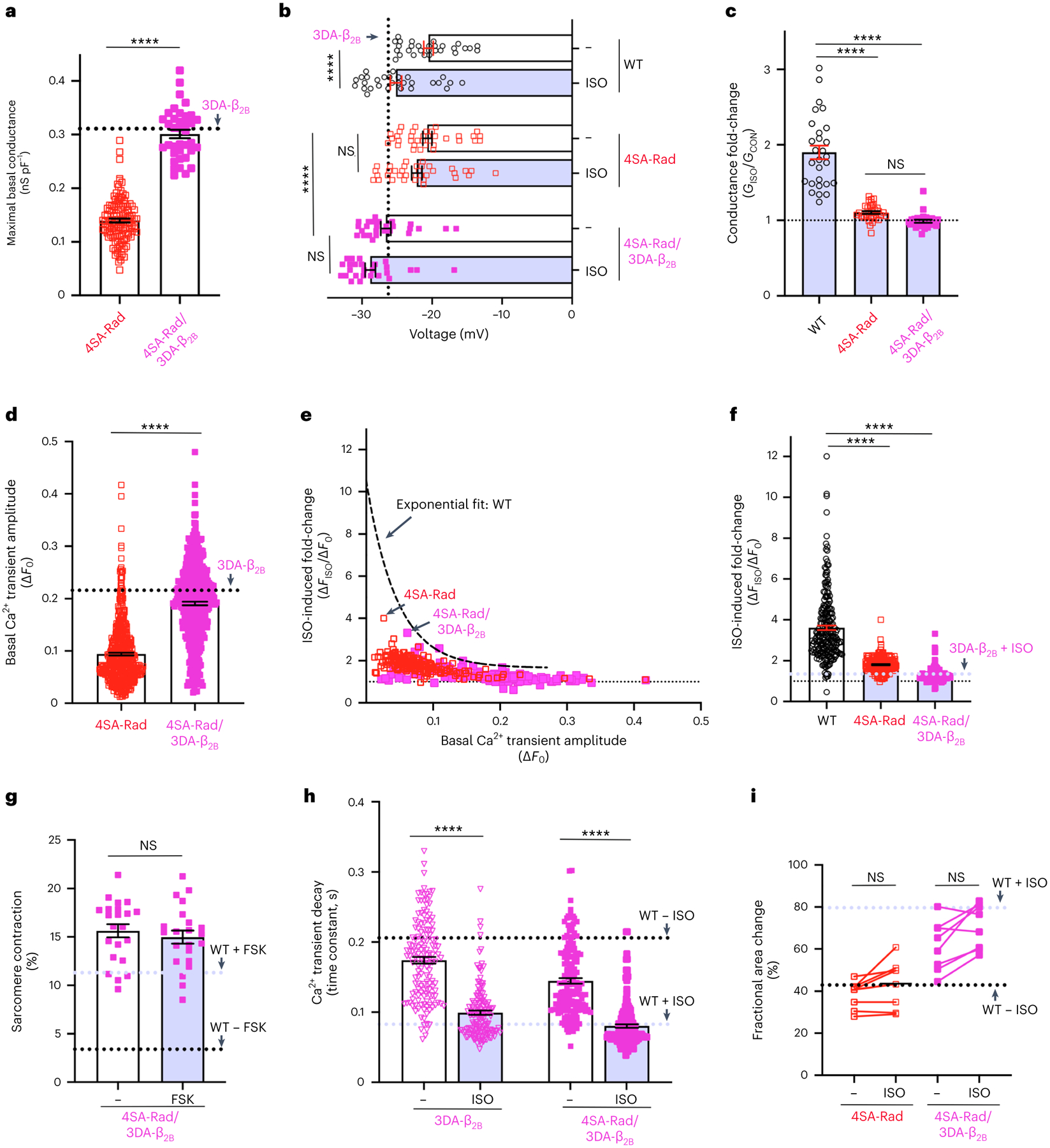
Adrenergic augmentation of contractility is CaV1.2 dependent. **a**, Maximal basal conductance (*G*_max_) density. The black dashed line is the mean 3DA-β_2B_ *G*_max_. Data are the mean ± s.e.m. ****P < 0.0001 unpaired, two-tailed Student’s *t*-test (*n* = 123 and 37 cells from 10 and 4 mice, from left to right). **b**, Graph of *V*_50_ acquired via ramp protocol. Data are mean ± s.e.m. *P* < 0.0001 by one-way ANOVA, ****P < 0.0001 by Sidak’s multiple-comparison test (*n* = 29, 29, 35, 35, 26, 26 cells from 3, 3, 3, 3, 4 and 4 mice, respectively, from top to bottom). **c**, Isoproterenol-induced fold-change of Ca^2+^ channel conductance at −20 mV, acquired via ramp protocol. Data are mean ± s.e.m. *P* < 0.0001 by one-way ANOVA, ****P < 0.0001 by Sidak’s multiple-comparison test (*n* = 28, 35 and 26 cells from 3, 3 and 4 mice, respectively, from left to right). **d**, Basal Ca^2+^ transient amplitude. Data are the mean ± s.e.m. ****P < 0.0001 by unpaired, two-tailed Student’s *t*-test (*n* = 507 4SA-Rad cells, 10 mice (same data as Fig. 3i); 467 4SA-Rad/3DA-β_2B_ cells, 4 mice). **e**, Isoproterenol-induced fold-change of Ca^2+^ transient amplitude versus basal amplitude. The black dashed line is the exponential fit for the WT (*n* = 183 4SA-Rad cells, 5 mice (same as Fig. 3d); 160 4SA-Rad/3DA-β_2B_ cells, 4 mice). **f**, Isoproterenol-induced fold-change in Ca^2+^ transient amplitude. The WT and 4SA-Rad data same as Fig. 3f. The dashed blue line is mean for isoproterenol-treated 3DA-β_2B_ cardiomyocytes. Data are the mean ± s.e.m. ****P < 0.0001 by unpaired, two-tailed Student’s *t*-test (*n* = 225, 183 and 160 cells from 4, 5 and 4 mice, respectively, from left to right). **g**, Change in sarcomere length. Data are the mean ± s.e.m. *P* = 0.50 by unpaired, two-tailed Student’s *t*-test (*n* = 22 cells, 3 mice). **h**, Time constant of Ca^2+^ reuptake. Data are the mean ± s.e.m. ****P < 0.0001 by unpaired, two-tailed Student’s *t*-test (*n* = 148 and 160 cells, from 3 and 4 mice, from left to right). **i**, Fractional area of change. 4SA-Rad is same data as Fig. 5k. Dashed lines are means for WT without and with isoproterenol. Data are mean ± s.e.m. *P* = 0.27 and *P* = 0.12 by unpaired, two-tailed Student’s *t*-test, from left to right (*n* = 8 mice for each group).

To verify that reduced basal Ca^2+^ influx and attenuated adrenergic agonist-induced augmentation of cardiac contractility in the 4SA-Rad mice are due to dysfunctional regulation of Ca^2+^ channels, we crossed the 4SA-Rad mice with 3DA-β_2B_ transgenic mice. As expected, cardiomyocytes isolated from combined homozygous 4SA-Rad and transgenic 3DA-β_2B_ mice had activated Ca^2+^ channels without exposure to adrenergic agonists, marked by increased conductance (Fig. 8a), hyperpolarized *V*_50_ for activation (Fig. 8b) and markedly attenuated adrenergic regulation (Fig. 8b,c). Furthermore, these myocytes displayed increased basal Ca^2+^ transients (Fig. 8d) and diminished adrenergic agonist-induced augmentation of both Ca^2+^ transients (Fig. 8e,f) and contractility (Fig. 8g). Isoproterenol accelerated the rate of Ca^2+^ reuptake (Fig. 8h), revealing that other adrenergic signaling pathways were unperturbed in these mice. The homozygous 4SA-Rad mice crossed with transgenic 3DA-β_2B_ mice displayed increased basal left ventricular contractility and diminished adrenergic-induced augmentation of contractility (Fig. 8i). As prevention of 4SA-Rad from binding to Ca_V_ β_2B_ increased the basal Ca^2+^ transient and contractility compared with 4SA-Rad alone, we exclude confounding ‘off-target’ functions and conclude that the phenotypes imparted by 4SA-Rad are solely due to a direct effect on Ca^2+^ channels. Moreover, that the Ca^2+^ current, Ca^2+^ transients and cardiac contractility can be augmented to near adrenergic-agonist levels independent of the β-adrenergic system and Rad phosphorylation suggests a therapeutic target for increasing cardiac contractility in patients with failing hearts.

## Discussion

We show that PKA phosphorylation of Rad is essential for regulation by the sympathetic nervous system of Ca^2+^ influx in atrial and ventricular myocytes and for augmentation of cardiac contractility. The adrenergic regulation of Ca_V_1.2 channels can be fully abrogated by preventing β-subunit binding to α_1C_^14^, introducing flexibility to the rigid linker^38^ between the β-subunit-binding site in the I–II loop and the channel pore^33^, replacing WT Rad with a mutant Rad that cannot be phosphorylated or preventing β-subunit binding to Rad. That no acute adrenergic increase in Ca^2+^ current is observed in any of these four distinct mouse lines suggests that plasma membrane insertion of additional Ca^2+^ channels after adrenergic stimulation^39^ is not a major contributor to augmentation of Ca^2+^ influx.

In the absence of Rad phosphorylation, adrenergic agonist-induced enhancement of cardiac contraction is almost completely disabled. As Rad-bound channels have very low open probability and are essentially electrically silent, Rad-less Ca^2+^ channels are the basis for the initiation of excitation–contraction coupling under basal conditions. The adrenergic reserve of Ca^2+^ influx and the potential to boost the contractile output, in contrast, are fully dependent on the Rad-bound Ca^2+^ channels in both the atrial and the ventricular chambers of the heart.

Is Rad phosphorylation and release of Ca^2+^ channel inhibition sufficient to independently elevate contractility? To answer this question, we developed mice with an ablated Rad–Ca_V_β interaction through mutations of Ca_V_β. Solely releasing the subpopulation of Rad-bound Ca^2+^ channels from inhibition without activating the β-adrenergic–PKA signaling pathways was sufficient to fully activate Ca^2+^ current, and to substantially augment the Ca^2+^ transient and contractility to the levels typically induced by adrenergic agonists. Our findings establish that the principal mechanism of adrenergic control of contractility is via enhancement of Ca^2+^ influx via Ca_V_1.2 channels. The several-fold increase in Ca^2+^ current results in increased triggering of RyR2 channels and increased Ca^2+^ release from the SR, leading to increased cardiac contractility. The small residual increase in the Ca^2+^ transient and contractility by adrenergic agonists in 4SA-Rad, 2DA-β_2B_ and 3DA-β_2B_ mice is probably due to phosphorylation of RyR2 (ref.^8^), PLN/SERCA^3,40^ and perhaps other targets.

Patients hospitalized with severely decompensated heart function have limited therapeutic options and are typically treated with invasive implantation of mechanical pumps, or β-adrenergic agonists or phosphodiesterase inhibitors, which increase PKA activity. Although the goals of these pharmacological interventions are to promote cardiac contractility^41^, their long-term use is limited by diminishing responses over time and substantial side effects in the cardiovascular and other organ systems^42,43^. We demonstrate that the disruption of the Rad–Ca_V_β interaction is an equally efficacious but substantially more specific downstream target than the currently available upstream cardiotherapeutic activators of the PKA signaling pathways. A therapeutic that targets this interaction would be the foundation for specific cardiac inotropes.

## Methods

### Generation of knock-in and transgenic mice

The present study conformed to the Guide for the Care and Use of Laboratory Animals of the National Institutes of Health (NIH) and protocols approved by the Institutional Animal Care and Use Committee of Columbia University. Animals were maintained under a standard 12-h light:12-h dark cycle and had free access to standard chow and water. We used male and female mice aged 6 weeks to 5 months. The investigators were blinded to group allocation during data acquisition and analysis.

The 4SA-Rad knock-in mouse line was generated by Genoway. The murine genomic region encompassing the targeted *Rrad* mouse gene from C57BL/6N mouse genomic (g)DNA was used for homologous recombination. Ser25 and Ser38 in exon 2 were mutated to alanine, and Ser272 and Ser300 in exon 5 were mutated to alanine. The LoxP/FRT Neo cassette in the intron between exons 4 and 5 was deleted through mating with C57BL/6N Cre-deleter mice (Extended Data Fig. 1a). The locus and a minimum of 1-kb downstream and upstream of each homology arm were sequenced. Mice were exclusively maintained in the C57BL/6N background by mating of heterozygous mice.

The WT and 3DA-β_2B_ transgenic mutant mouse constructs were created by ligating in-frame a 3× FLAG epitope to the amino-terminus of human *CACNB2b* cDNA (accession no. AAG01473) and mutating residues Asp244, Asp320 and Asp322 to alanine by site-directed mutagenesis. The WT and the 3DA-β_2B_ cDNAs were ligated into the pJG/α-myosin heavy chain (MHC) plasmid (a gift from J. Robbins, Addgene plasmid no. 55594)^44^, between the 5.5-kb murine α-MHC promoter and the human growth hormone polyadenylation sequence. The transgenic mice were created by the Genetically Modified Mouse Models Shared Resource at Columbia University. The WT and the 3DA-β_2B_ transgenic mice, on a B6CBA/F2 hybrid background, were bred with WT C57BL/6N mice or 4SA-Rad mice, which are on the C57BL/6N background.

The 2DA-β_2B_ knock-in mouse line, with alanine substitutions for the two aspartate residues in exon 11 (equivalent of human Asp320 and Asp322), was created using CRISPR–Cas9 gene editing. Validation of the single guide (sg)RNA and single-strand oligodeoxynucleotide (ssODN) was performed in the Genome Engineering and iPSC Center (GEic) at Washington University (Extended Data Fig. 5c). Zygotes isolated from C57BL/6N mice were electroporated with the sgRNA and ssODN at Mount Sinai School of Medicine Mouse Genetics and Gene Targeting Core. Identification of potential founders and germline transmission after crossing with WT C57BL/6N mice was performed at Washington University by deep sequencing of gDNA from tail biopsies. Heterozygous 2DA-β_2B_ offspring mice were crossed to obtain homozygotes. Genotypes were identified by PCR of gDNA and sequencing.

### Histology

Total body weight and tibial length were measured for 2- to 9-month-old mice. Hearts and lungs were harvested and weighed. Hearts were fixed in 4% paraformaldehyde overnight and processed for routine paraffin histology. They were stained with hematoxylin and eosin and Masson’s trichrome.

### Cellular electrophysiology

Mice ventricular myocytes were isolated by enzymatic digestion using a Langendorff perfusion apparatus^13,14,45–47^. Isolated cardiomyocytes were placed in Petri dishes filled with solution containing 112 mM NaCl, 5.4 mM KCl, 1.7 mM NaH_2_PO_4_, 1.6 mM MgCl_2_, 20.4 mM Hepes, pH 7.2, 30 mM taurine, 2 mM dl-carnitine, 2.3 mM creatine and 5.4 mM glucose. The pipette resistance was between 0.5 MΩ and 1.5 MΩ. The pipette solution contained 40 mM CsCl, 80 mM cesium gluconate, 10 mM 1,2-bis(*o*-aminophenoxy)ethane-*N*,*N*,*N*′, *N*′-tetraacetic acid (BAPTA), 1 mM MgCl_2_, 4 mM Mg ATP and 10 mM Hepes, adjusted to pH 7.2 with CsOH. After the isolated cardiomyocytes were dialyzed and adequately buffered with 10 mM BAPTA in the internal solution, cells were locally superfused with 140 mM tetraethylammonium chloride, 1.8 mM CaCl_2_ (or 0.5 mM BaCl_2_), 1 mM MgCl_2_, 5 mM glucose and 10 mM Hepes, adjusted to pH 7.4 with CsOH. To measure peak currents, we held the cell membrane potential at −60 mV and stepped it to +50 mV for 150 ms in 10-mV increments every 10 s. Cells without a stable baseline (possibly due to run-down or run-up) were not studied. Membrane currents were measured by the conventional whole-cell patch-clamp method using a MultiClamp 700B amplifier and pCLAMP 10.7 software (Molecular Devices). The acquisition sampling rate for this step protocol was 20 kHz. Capacitance transients and series resistance were compensated for (>85%). Voltage was corrected for liquid junction potential (−10 mV) during analysis. Leak currents were subtracted by a P/3 protocol. The conductance was normalized to cell size. The voltage-step protocol used in cardiomyocytes studies evaluated *I*_peak_ = *I*_peak_(*V*), which was recalculated in CLAMPFIT to *G* = *G*(*V*) as *G* = *I*/(*V* − *E*_rev_). The parameters of voltage-dependent activation were obtained using a Boltzmann approximation curve^15^.

In many experiments, we used a ramp protocol with a 200-ms voltage ramp from −60 mV to +60 mV (0.6 V s^−1^) applied every 6 s to monitor the current–voltage (*I*–*V*) relationship. Voltage was corrected for liquid junction potential (10 mV) during analysis. Leak currents were subtracted by a P/3 protocol. Capacitance transients and series resistance were compensated for (>85%). The acquisition sampling rate for the ramp protocol was 5 kHz. In these experiments the external solution contained 0.5 mM BaCl_2_ instead of 1.8 mM CaCl_2_. Under these conditions, currents through Ca^2+^ channels are small (<1 nA) and showed practically no inactivation. After establishing stable records (usually after 2–3 min), 10–15 traces were recorded for the control. Thereafter, isoproterenol or forskolin was superfused. After the response stabilized, typically within 2–3 min for isoproterenol and 3–6 min for forskolin, 10–15 additional traces were recorded. When no response was observed, we continued the experiments for 4–6 min. The 10–15 traces for control and post-isoproterenol or forskolin were averaged. We transformed *I* = *I*(*t*) to *I* = *I*(*V*), which was then further recalculated to *G* = *G*(*V*) in CLAMPFIT. The parameters of voltage-dependent activation in control and post-isoproterenol or forskolin were obtained by fitting with the Boltzmann function in Prism (GraphPad) or MATLAB, with a goodness of fit (*R*^2^) of >0.999. The data were imported into Origin (v.7.5) for generations of figures.

Forskolin (Santa Cruz, catalog no. sc-3562) was used at a concentration of 10 μM made from a 10-mM stock dissolved in dimethylsulfoxide. Isoproterenol (Sigma-Aldrich, catalog no. I5627) was applied at a concentration of 200 nM. Ethylenediaminetetraacetic acid (EDTA), 50 μM, was added to prevent fast degradation of isoproterenol^48^. Calyculin A (LC Laboratories, catalog no. C-3987) was used at a concentration of 100 nM made from a 100-μM stock.

### Immunoprecipitation and western blotting

In some experiments, cardiomyocytes were incubated with either 1 μM isoproterenol or 10 μM forskolin before lysis. Cardiomyocytes were lysed with a hand-held tip homogenizer in a 1% (v:v) Triton X-100 buffer containing: 50 mM Tris-HCl, pH 7.4, 150 mM NaCl, 10 mM EDTA, 10 mM ethylene glycol-bis(β-aminoethyl ether)-*N*,*N*,*N*′,*N*′-tetraacetic acid (EGTA), Complete Mini Protease inhibitor tablet (Roche) and PhosSTOP (Roche). The lysates were incubated on ice for 30 min, centrifuged at 21,130*g* and 4 °C for 10 min and supernatants collected. Immunoprecipitation of Ca_V_1.2 complexes was performed with a customized rabbit polyclonal anti-α_1C_-subunit antibody (Yenzym). Immune complexes were collected using protein A (Amersham) for 2 h, followed by extensive washing. For western blotting, proteins were size separated on sodium dodecylsulfate–polyacrylamide gel electrophoresis, transferred to nitrocellulose membranes and probed with either the customized rabbit polyclonal anti-α_1C_-subunit antibody (1:1,000) or a guinea-pig anti-α_1C_ antibody (Alomone, catalog no. AGP-001, 1:400)^49,50^, a customized polyclonal anti-β-subunit antibody (epitope: mouse residues 120–138: DSYTSRPSDSDVSLEEDRE; Yenzym, 1:1,000), anti-SERCA2 antibody (Alomone, catalog no. ACP-012, 1:1,000), anti-NCX1 antibody (Alomone, catalog no. ANX-011, 1:1,000), a customized polyclonal anti-Rad antibody (epitope: GSRGAGRERDRRRG, Yenzym, 1:1,000), anti-β-actin antibody (Santa Cruz, catalog no. sc-47778, 1:1,000), a customized anti-RyR2 antibody (1:5,000)^7^, a customized anti-Ser2808 RyR2 antibody (1:5,000, gift of A. Marks)^8^, an anti-Ser2808 RyR2 antibody (Thermo Fisher Scientific/Invitrogen, catalog no. PA5–105712, 1:1,000), anti-PLN antibody (Cell Signaling, catalog no. 14562, 1:1,000), an anti-phospho-PLN (Ser16/Thr17) antibody (Cell Signaling, catalog no. 8496, 1:1,000), an anti-Ser23/Ser24 TnI antibody (Phosphosolutions, catalog no. p2010–2324, 1:1,000), an anti-TnI antibody (Phosphosolutions, catalog no. 2010-TnI, 1:2,000) and an anti-FLAG antibody (Sigma-Aldrich, catalog no. F7425, 1:1,000). Signal detection was performed with goat anti-mouse horseradish-peroxidase (HRP)-conjugated secondary antibody (Thermo Fisher Scientific, catalog no. 31430, 1:5,000), goat anti-rabbit HRP-conjugated secondary antibody (Thermo Fisher Scientific, catalog no. G21234, 1:5,000), goat anti-guinea-pig HRP-conjugated secondary antibody (Thermo Fisher Scientific, catalog no. A18775, 1:5,000) and enhanced chemiluminescence using an Azure Biosystem 600 imager. The intensity of the signals was quantified using ImageJ.

### RNA-seq

Mouse hearts were removed, the atria removed and the ventricles snap-frozen in liquid nitrogen before storing at −80 °C. RNA-seq was performed at the Genomics and High Throughput Screening Shared Resource at Columbia University. RNA was extracted from the samples with QIAGEN miRNeasy micro kit (catalog no. 217084) following the kit protocol, except 0.7 volume of 100% ethanol was used instead of 1.5 volume of 100% ethanol for binding of total RNA on to the column. We used poly(A) pull-down to enrich mRNAs from total RNA samples and then proceeded with library construction using Illumina TruSeq chemistry. Libraries were then sequenced using Illumina NovaSeq 6000. We multiplexed samples in each lane, which yielded targeted number of paired-end 100-bp reads for each sample. Real-time analysis (Illumina) was used for base calling and bcl2fastq2 (v.2.19) was used for converting BCL to fastq format, coupled with adapter trimming. We performed a pseudoalignment to a kallisto index created from transcriptomes (GRCm38) using kallisto (0.44.0). Differential gene expression analysis in the 4SA-RAD knock-in versus littermate control mice was performed using the R package DESeq2 (v.1.13.0) from unnormalized count matrix with a false discovery rate (FDR) cut-off of 0.05.

### Fractional shortening of isolated cardiomyocytes

Freshly isolated myocytes were superfused with Tyrode’s solution containing 1.2 mM CaCl_2_. Myocytes were field stimulated at 1 Hz. The percentage contraction of the sarcomere length was measured using the SarcLen module of Ionoptix and calculated as the difference of shortest sarcomere length during a contraction subtracted from the relaxed sarcomere length, divided by the relaxed sarcomere length, all averaged over at least eight contractions.

### Calcium imaging

Cells were incubated at a final concentration of 2.5 μM Fura-2AM (Invitrogen, catalog no. F1221) for 15 min. The cells were washed several times with Ringer’s solution containing: 112 mM NaCl, 5.4 mM KCl, 1.7 mM NaH_2_PO_4_, 20.4 mM Hepes, 30 mM taurine, 2 mM dl-carnitine, 2.3 mM creatine, 5.4 mM glucose, 1.6 mM MgCl_2_, 1.2 mM CaCl_2_ and 10 mM 2,3-butanedione, pH 7.2. Cells were plated on to coverslips coated with laminin (Sigma-Aldrich, catalog no. L2020). Coverslips in Bioptechs Delta T dishes, which served as the perfusion chamber, were placed on the stage of a Nikon Eclipse Ti inverted microscope. Cardiomyocytes were locally perfused with Tyrode’s solution containing 134 mM NaCl, 5.4 mM KCl, 1.2 mM CaCl_2_, 1.0 mM MgCl_2_, 10 mM Hepes and 10 mM glucose. Cardiomyocytes were field stimulated at 1 Hz for the duration of the experiment except for 5 s before and during infusion of caffeine. Fluorescence excitation was via a 340-nm and 380-nm LED illumination system (pE-340^fura^, CoolLED). Emission was detected at 510 nm through a Nikon Fluor ×10 objective using a Prime BSI-Express sCMOS (scientific Complementary Metal–Oxide–Semiconductor) camera (Teledyne Photometrics) and Nikon Elements (v.5-21-03). The emission due to excitation by 340 nm (*F*_340_) was acquired for 20 ms and the emission due to excitation by 380 nm (*F*_380_) was subsequently acquired for 20 ms. To minimize bleaching, however, fluorescence was acquired for only 10 s before superfusion of isoproterenol or forskolin, for 10 s after a 2-min superfusion of isoproterenol (100 nM) or forskolin (5 μM) and for 30 s during the superfusion of 10 mM caffeine at the conclusion of the experiment. The entire experiment (perfusion, recording and pacing) was automated using Nikon Elements, STV-2–4MX-1 valve (Takasago) and MyoPacer (IonOptix) to eliminate user variability during acquisition.

After the acquisition, cardiomyocytes were demarcated using Nikon Elements software. An area was selected without cardiomyocytes for background subtraction. Analysis was completed using a customized MATLAB (R2021b) script, which identified the basal diastolic fluorescence ratio (*F*_340_:*F*_380_) and the pacing-induced Ca^2+^ transient, Δ*F* (difference of *F*_340_:*F*_380_ ratio of basal), before pacing (diastole) and at the peak after field stimulation (systole) for both pre- and post-infusion of isoproterenol or forskolin for the middle seven to eight of ten acquired transients. The time of fluorescence signal decay (*t*) to the levels of 37% peak for each transient was calculated in MATLAB and used to determine intracellular Ca^2+^ decay kinetics. Δ*F*_caffeine_ was the difference of peak *F*_340_:*F*_380_ post-caffeine versus basal *F*_340_:*F*_380_ pre-caffeine, which is proportional to SR Ca^2+^ load.

### Echocardiograms

Mice, aged 6–20 weeks, were anesthetized with 1–2% inhalational isoflurane and transthoracic echocardiography was performed using a 25- to 55-MHz linear-array transducer probe with a digital ultrasound system (Vevo 3100 Image System, VisualSonics). Vevo LAB 3.0 ultrasound analysis software (Fujifilm, VisualSonics) was used to measure and analyze images. Each echocardiographic parameter is the average of two measurements obtained from different cines. Vevo Strain speckle tracking and analysis software were used to calculate left ventricular strain values by the formula ((*l*_1_ − *l*_0_)/*l*_0_) × 100. Ejection fraction was calculated in Vevo Strain using Simpson’s biplane method. GLS was calculated from an apical four-chamber view as follows: GLS = (*L*_(t)_ − *L*_0_)/*L*_0_, where *L*_0_ is the length of the heart in end-diastole. GCS was similarly calculated from a parasternal short-axis view using circumference rather than length. In a subset of mice, 2 mg kg^−1^ of isoproterenol via intraperitoneal injection was administered and echocardiograms were reacquired 2–5 min post-injection.

### Telemetry and ECG analysis

Telemetry devices (Data Sciences International, model ETA-F10) were implanted in 6- to 10-week-old mice. Recordings were begun 1 week after implantation. Intervals were measured using Ponemah software. Mice were housed in individual cages after telemeter implantation and for the entire experiment, on 12-h light:12-h dark cycle. Saline (control) or 2 mg kg^−1^ of isoproterenol dissolved in saline was administered via intraperitoneal injection. ECGs were recorded both before and after injections. Heart rate values were averaged over 3-min time periods.

### Exercise treadmill testing

Exercise testing was performed in the Mouse NeuroBehavior Core of the Institute of Genomic Medicine at Columbia University using established protocols. Male and female mice, aged 2–4 months, were acclimated to the treadmill (Panlab/Harvard Apparatus Treadmill) for 2 d (training). During training, the speed of the treadmill was gradually increased from 5 cm s^−1^ to 20 cm s^−1^ for up to 20 min, unless mice failed. Mice were considered to have failed the training if they spent 5 s consecutively in the ‘fatigue zone’, defined as one body length area toward the end of the belt, and/or if they received more than ten shocks. After training, the mice were challenged in a more difficult trial. On the challenge day, mice were run on the treadmill with no incline at 25 cm s^−1^ for 20 min. Failure to complete the exercise was based on two criteria: if mice remained in the fatigue zone for 5 s consecutively or if they received >20 shocks. For the progressive speed/incline study, mice were run on the treadmill with an incline of 15° with increasing speed from 10 cm s^−1^ to 30 cm s^−1^ (increase of 5 cm s^−1^ every 2 min) for up to 20 min. The definition of failure to complete the challenge was the same as for the flat incline/constant speed.

### Flow cytometric FRET two-hybrid assay

HEK293T cells (American Type Culture Collection, catalog no. CRL-3216) were cultured in 12-well plates and transfected with Lipofectamine 2000 (Thermo Fisher Scientific, catalog no. 11668019). Cer-WT β_2B,_ 3DA-β_2B_ or 2DA-β_2B_ and Ven-tagged Rad, Ven-tagged 4SA-Rad or Ven-tagged I–II loop cDNA pairs (1 μg) were mixed in serum-free Dubecco’s modified Eagle’s medium. FRET experiments were performed 1 d post-transfection. The protein-synthesis inhibitor cycloheximide (100 μM) was added to cells 2 h before experimentation to halt synthesis of new fluorophores and allow existing fluorophores to fully mature. For FRET measurements, we used an LSR II (BD Biosciences) flow cytometer, equipped with 405-nm, 488-nm and 633-nm lasers for excitation and 18 different emission channels. For calyculin experiments (Fig. 2), calyculin A was added at a final concentration of 100 nM for 10 min. The FRET analysis software is accessible on github at https://github.com/manubenjohny/FACS_FRET.

### Proximity proteomics and MS

Proximity labeling was performed^51^ with minor modifications^15,52^. Isolated ventricular cardiomyocytes from mice expressing rabbit α_1C_-APEX2 were incubated in labeling solution with 0.5 μM biotinphenol (Iris-biotech) for 30 min. During the final 10 min of labeling, 1 μM isoproterenol or 100 nM calyculin A was added. To initiate labeling, H_2_O_2_ (Sigma-Aldrich, catalog no. H1009) was added to a final concentration of 1 mM. Exactly 1 min after H_2_O_2_ treatment, the labeling solution was decanted and cells were washed 3× with cold quenching solution containing 10 mM sodium ascorbate (VWR 95035–692), 5 mM Trolox (Sigma-Aldrich, catalog no. 238813) and 10 mM sodium azide (Sigma-Aldrich, catalog no. S2002). After cells were harvested by centrifugation, the quenching solution was aspirated, and the pellet was flash-frozen and stored at −80 °C until streptavidin pull-down.

Subsequent protein processing procedures and MS analysis were performed as described^15,53–56^. The digested peptides were labeled with TMTpro 16-plex (Thermo Fisher Scientific, catalog no. A44520) for 1 h. Data collection followed a MultiNotch MS^3^ TMT method^57^ using an Orbitrap Lumos mass spectrometer coupled to a Proxeon EASY-nLC 1200 liquid chromatography system (both Thermo Fisher Scientific)^15,55^. Peptides were searched with SEQUEST (v.28, rev. 12)-based software against a size-sorted forward and reverse database of the *Mus musculus* proteome (Uniprot 07/2014) with added common contaminant proteins and rabbit α1C sp|P15381|CAC1C_RABIT. For this, spectra were first converted to mzXML. Searches were performed using a mass tolerance of 20 p.p.m. for precursors and a fragment ion tolerance of 0.9 Da. For the searches maximally two missed cleavages per peptide were allowed. Carboxyamidomethylation on cysteine was set as a static modification (+57.0214 Da) and we searched dynamically for oxidized methionine residues (+15.9949 Da). We applied a target decoy database strategy and an FDR of 1% was set for peptide–spectrum matches after filtering by linear discriminant analysis^55,58^. The FDR for final collapsed proteins was 1%. MS^1^ data were calibrated post-search and searches performed again. Quantitative information on peptides was derived from MS^3^ scans. Quantitative tables were generated requiring an MS^2^ isolation specificity of >70% for each peptide and a sum of TMT (tandem mass tags) signal:noise ratio (s:n) of >200 over all channels for any given peptide, and then exported to Excel and further processed therein. Proteomics raw data and search results were deposited in the PRIDE archive^59,60^. The relative summed TMT s:n for proteins between two experimental conditions was calculated from the sum of TMT s:n for all peptides of a given protein quantified.

### Statistics

The results are presented as the mean ± s.e.m. For multiple-group comparisons, a one-way analysis of variance (ANOVA) followed by multiple-comparison testing were performed. For comparisons between two groups, an unpaired, two-tailed Student’s *t*-test was used. Statistical analyses were performed using Prism 8 (Graphpad). Differences were considered statistically significant at values of *P* < 0.05.

### Reporting summary

Further information on research design is available in the Nature Research Reporting Summary linked to this article.

## Extended Data

## Supplementary Material

Supplemental Data Table 1

Supplemental Data Table 2

## Data Availability

RNA-seq data have been uploaded to the Gene Expression Omnibus (accession no. GSE198903). Proteomics raw data and search results were deposited in the PRIDE archive and can be accessed via the ProteomeXchange under accession no. PXD033492. All other data are available in the main text and related files. Source data are provided with this paper.
